# Analysis of the main antioxidant enzymes in the roots of *Tamarix ramosissima* under NaCl stress by applying exogenous potassium (K^+^)

**DOI:** 10.3389/fpls.2023.1114266

**Published:** 2023-04-18

**Authors:** Yahui Chen, Haijia Li, Shiyang Zhang, Shanfeng Du, Jinchi Zhang, Zhizhong Song, Jiang Jiang

**Affiliations:** ^1^ Collaborative Innovation Center of Sustainable Forestry in Southern China of Jiangsu Province, Nanjing Forestry University, Nanjing, China; ^2^ Jiangsu Academy of Forestry, Nanjing, China; ^3^ The Engineering Research Institute of Agriculture and Forestry, Ludong University, Yantai, China; ^4^ Faculty of science and Department of statistic, University of British Columbia, Vancouver, BC, Canada

**Keywords:** metabolomic, NaCl stress, NaCl toxicity, potassium (K^+^), *Tamarix ramosissima*; transcriptomic

## Abstract

**Introduction:**

Salinization affects more than 25% of the world's arable land, and *Tamarix ramosissima* Ledeb (*T. ramosissima*), the representative of *Tamarix* plants, is widely grown in salinized soil. In contrast, less is known about the mechanism of potassium's antioxidative enzyme activity in preventing NaCl stress damage to plants.

**Method:**

This study examined changes in root growth for *T. ramosissima* at 0h, 48h, and 168h, performed antioxidant enzyme activity assays, transcriptome sequencing, and non-targeted metabolite analysis to understand changes in their roots as well as changes in the activities of superoxide dismutase (SOD), peroxidase (POD), and catalase (CAT). Quantitative real-time PCR (qRT-PCR) was used to identify differentially expressed genes (DEGs) and differential metabolites associated with antioxidant enzyme activities.

**Result:**

As the time increased, the results showed that compared with the 200 Mm NaCl group, the root growth of the 200 mM NaCl + 10 mM KCl group increased, the activities of SOD, POD and CAT increased the most, but the contents of hydrogen peroxide (H2O2) and Malondialdehyde (MDA) increased less. Meanwhile, 58 DEGs related to SOD, POD and CAT activities were changed during the application of exogenous K+ for 48h and 168h in *T. ramosissima*. Based on association analysis of transcriptomic and metabolomic data, we found coniferyl alcohol, which can act as a substrate to label catalytic POD. It is worth noting that *Unigene0013825* and *Unigene0014843*, as POD-related genes, have positively regulated the downstream of coniferyl alcohol, and they have a significant correlation with coniferyl alcohol.

**Discussion:**

In summary, 48h and 168h of exogenous K^+^ applied to the roots of *T. ramosissima* under NaCl stress can resist NaCl stress by scavenging the reactive oxygen species (ROS) generated by high salt stress by enhancing the mechanism of antioxidant enzyme activity, relieving NaCl toxicity and maintaining growth. This study provides genetic resources and a scientific theoretical basis for further breeding of salt-tolerant *Tamarix* plants and the molecular mechanism of K^+^ alleviating NaCl toxicity.

## 1 Introduction

There is a wide distribution of saline soils throughout the world. The high salinity content and poor physical and chemical properties of saline soil made it difficult for plants to grow and develop. The most widely distributed salt within the saline soil is NaCl. As plants grow and develop, it can negatively affect several physiological and biochemical processes (Negrão et al., 2017). Crop varieties which have been bred with salt tolerance characteristics have made considerable progress, yet most varieties produced in saline-alkaline soils still have low yields. The rational improvement and utilization of saline soils has therefore become an urgent issue that needs to be addressed (Rahman et al., 2021).

Salt stress is one of the most important abiotic stresses that seriously threaten the sustainability of agroforestry. Salt stress is a complex mechanism that affects almost all physiological pathways of plant growth and development (Cuartero et al., 2006; Nabati et al., 2011). In salt stress, plants’ root systems are the first organs to feel the stress signal, and they are most directly affected. (Kenrick and Crane, 1997; Duan et al., 2022; Yang et al., 2022). Under normal plant growth conditions, the root system can absorb water and nutrients to maintain the dynamic balance of cells (Jbir et al., 2001; Ibrahim et al., 2017), but salt stress will inhibit plant root respiration, affect root metabolism, and lead to internal root dysfunction, thereby destroying this balance (Costa et al., 2007). Meanwhile, salt stress may lead to the disintegration of membranes in plants, the production of toxic metabolites, the inhibition of photosynthesis, the production of ROS, and the weakness of nutrient acquisition, which may result in cell break and plant death (Hasegawa et al., 2000; Ashraf, 2004; Chartzoulakis and Psarras, 2005; Sun et al., 2011). The main types of ROS are hydrogen peroxide (H_2_O_2_). In the case of an excess accumulation of ROS in the membrane system, membrane lipids will become peroxidized, causing malondialdehyde (MDA) to bind to proteins and deactivate them. When ROS is overproduced, plants cannot grow normally and even die if they are exposed to too much ROS. (Zhang et al., 2012). Reducing or eliminating the effect of ROS on plant cells is one of the important procedures to improve plant salt tolerance. Higher plants developed complex regulatory mechanisms during the long-term evolution to maintain ROS homeostasis. Notably, ROS generated by plants under salt stress can activate enzymatic and non-enzymatic systems to alleviate oxidative stress. The role of the enzymatic and non-enzymatic systems is mainly to eliminate ROS, reduce its damage to plant cells, and improve the antioxidant capacity of plants (Zhu et al., 2021; Chen et al., 2022b). In addition, the Na^+^ that plants are forced to absorb under high-salt stress conditions gradually accumulates, which inhibits the uptake of K^+^ by plants. A lack of K^+^ will reduce plant growth and productivity, and it is necessary to maintain the K^+^/Na^+^ ratio balance. Among various macronutrients, K^+^ plays an important role in the survival of plants under salt stress conditions (Mahmood, 2011), and a well-balanced K^+^/Na^+^ ratio is essential for the proper regulation of stomatal function, activation of enzymes, protein synthesis, cell osmoregulation, oxidant metabolism, photosynthesis, and maintenance of plant growth (Abbasi et al., 2014). Therefore, plants have to maintain a sufficient amount of K^+^ in the cytoplasm to improve plant growth and salt tolerance.

Belonging to the halophilic halophyte, *Tamarix ramosissima* Ledeb (*T. ramosissima*) is highly tolerant to various abiotic stresses, such as salt, drought and high temperature after long-term survival and evolution (Ramadan, 1998). *T. ramosissima* has developed a highly efficient abiotic stress tolerance system to adapt to adverse environments (Wang et al., 2021), including salt avoidance by roots and salt secretion by salt glands. In particular, *Tamarix* plants can reject 83% to 95% of the salt *in vitro* (Ramadan, 1998). In addition, halophytes have the ability to retain more K^+^ under salt stress conditions (Garthwaite et al., 2005). Their uptake and transport of K^+^ depend on various K^+^ transport proteins to adapt to different saline-alkali requirements. The high demand for K^+^ for halophytes requires efficient uptake from soil solutions by roots and further transfer to the above-ground parts, intracellular distribution to different compartments, and fulfillment by various K^+^ (Na^+^) transport systems. Moreover, a low concentration (≤ 100 mM) of NaCl stress can promote the growth of *T. ramosissima*, while a high concentration (≥ 200 mM) of NaCl inhibits its growth (Lu et al., 2014). The addition of exogenous 10 mM KCl can effectively alleviate the toxic effects of drought stress on the growth of *Alternanthera philoxeroides*, enhance the K^+^ enrichment level in plants (Song and Su, 2012), and overexpression of a K^+^ transporter gene, *ApKUP4*, in Arabidopsis thaliana significantly enhanced plant tolerance to NaCl stress by strengthening the K^+^ enrichment status and ROS scavenging capacity of transgenic plants (Song et al., 2014).

In this study, we combined physiological, transcriptomic, and metabolomic data to analyze the main antioxidant enzyme activities of *T.ramosissima*. This study provides a theoretical basis for revealing the molecular mechanism of plant tolerance to salt toxicity and contributes to the breeding of salt-tolerant plants.

## 2 Materials and methods

### 2.1 Material selection

*T. ramosissima* plants were donated by the Dongying Experimental Base of Shandong Academy of forestry sciences. experiments were carried out in the key laboratory, faculty of forestry, Nanjing Forestry University, from October 2019 to May 2021. 5-month-old *T. ramosissima* cuttings with similar growth were collected and transferred to a 24-well hydroponic box (40 cm × 30 cm × 16 cm in size) filled with 1/2 Hoagland nutrient solution and then placed in a greenhouse with a temperature of 26 ± 2°C and relative humidity of 40% to 55% for 2 months before analysis. The culture solution was changed every 3 days.

### 2.2 NaCl treatment

1/2 Hoagland nutrient solution was used as a control group. 1/2 Hoagland nutrient solution supplemented with 200 mM NaCl and 1/2 Hoagland nutrient solution of 200 mM NaCl +10mM KCl were cultured as the treatment groups, and the culture solution was changed every 3 days. After the root samples were collected at 0 h, 48 h, and 168 h after treatment, they were immediately treated with liquid nitrogen and stored at –80°C. Three repeats of eight seedlings were carried out for each treatment.

### 2.3 Changes in the growth of the roots of T. ramosissima

We collected samples exposed to each treatment at 0d, 48h and 168h, and photographed with a Leica D-LUX7 (Leica, Hesse, Germany) digital camera to observe the root changes and analyze them. A ruler was used (scale accurate to 0.1 cm) to measure the root length of the *T. ramosissima* at 0 h, 48 h and 168 h within the control group, 200 mM NaCl group, and 200 mM NaCl +10 mM KCl group.

### 2.4 Determination of antioxidant enzyme activity, H_2_O_2_ and MDA content

We selected 3 time points of 0h, 48h and 168h in the control group, 200 mM NaCl group and 200 mM NaCl+ 10 mM NaCl group, treating 24 *T. ramosissima* seedlings each time. We selected 8 *T. ramosissima* plants per time point for root sample collection for experimental determination, and biological replications were performed three times, for a total of 24 *T. ramosissima* seedlings. Their root tissues were sampled to determine the superoxide dismutase (SOD) activity, peroxidase (POD) activity, catalase (CAT) activity (Chen et al., 2022b), and H_2_O_2_ (Eisenberg, 1943) and MDA (Davey et al., 2005) content of *T. ramosissima* under different treatments. 3 biological replicates were applied for all determination.

### 2.5 Transcriptome sequencing and differentially expressed genes(DEGs)screening

After treatment with liquid nitrogen, *T. ramosissima* root samples were sent to the Guangzhou GENE Denovo Company (GENE Denovo, Guangzhou, China) for 3rd generation of high-throughput transcriptome sequencing. We uploaded the obtained transcriptome data to the National Center for Biotechnology Information (NCBI) Short Reads Archive (SRA) database with accession number SRP356215. Then, we followed the method of Chen et al. (Chen et al., 2022a) to perform gene ontology (GO) enrichment, the Kyoto Encyclopedia of Genes and Genomes (KEGG) annotation analysis, and DEG screening.

### 2.6 Metabolic extraction, detection and differential metabolic screening

After treatment with liquid nitrogen, *T. ramosissima* root samples were sent to the Guangzhou GENE Denovo Company (GENE Denovo, Guangzhou, China) for metabolite extraction and detection. Then, we performed Liquid chromatography–mass spectrometry (LC-MS) analysis and differential metabolite screening on the obtained metabolites following the method of Chen et al. (Chen et al., 2022c).

### 2.7 Quantitative Real-Time PCR validation of DEGs

8 key DEGs were randomly selected to verify the accuracy of RNA-Seq results. Total RNA was extracted from the root samples of the control and treatment groups using an Omega kit (Beinuo Bio, China), and the RNA was reverse transcribed into cDNA using the kit PrimerScript™ RT Master Mix (Perfect Real Time) (Dalian Bao Bio, China). Primers for key DEGs were designed and detected by qRT-PCR (**Supplementary Table S5**). Using the cDNA of the root tissue samples obtained by reverse transcription as a template, using PowerUp™ SYBR Green Master mix reagent (Thermo Fisher, China), the target gene qRT-PCR detection was carried out on the platform of ABI ViiA™ 7 Real-time PCR system (ABI, USA) -PCR detection. *Tubulin* was used as the internal reference gene, and the relative expression was calculated by the 2^−ΔΔCt^ method (Chen et al., 2022a).

### 2.8 Experiment processing

In this study, we used Excel (Microsoft, Washington, USA) to process all the data, used SPSS 26.0 (IBM, Chicago, USA) to perform significant analysis, utilized Origin 2018 software (Origin Lab Corporation, Northampton, USA) to make the graph, and used MEGA 11 software (MEGA Software, Pennsylvania, USA) to create phylogenetic trees. Then, we used ANOVA for significance testing, transcriptome sequencing and metabolite detection were repeated 3 times technically and 3 times biologically.

## 3 Results

### 3.1 Effects of NaCl stress on the roots of T. ramosissima with exogenous K^+^ application

**Figure 1:**
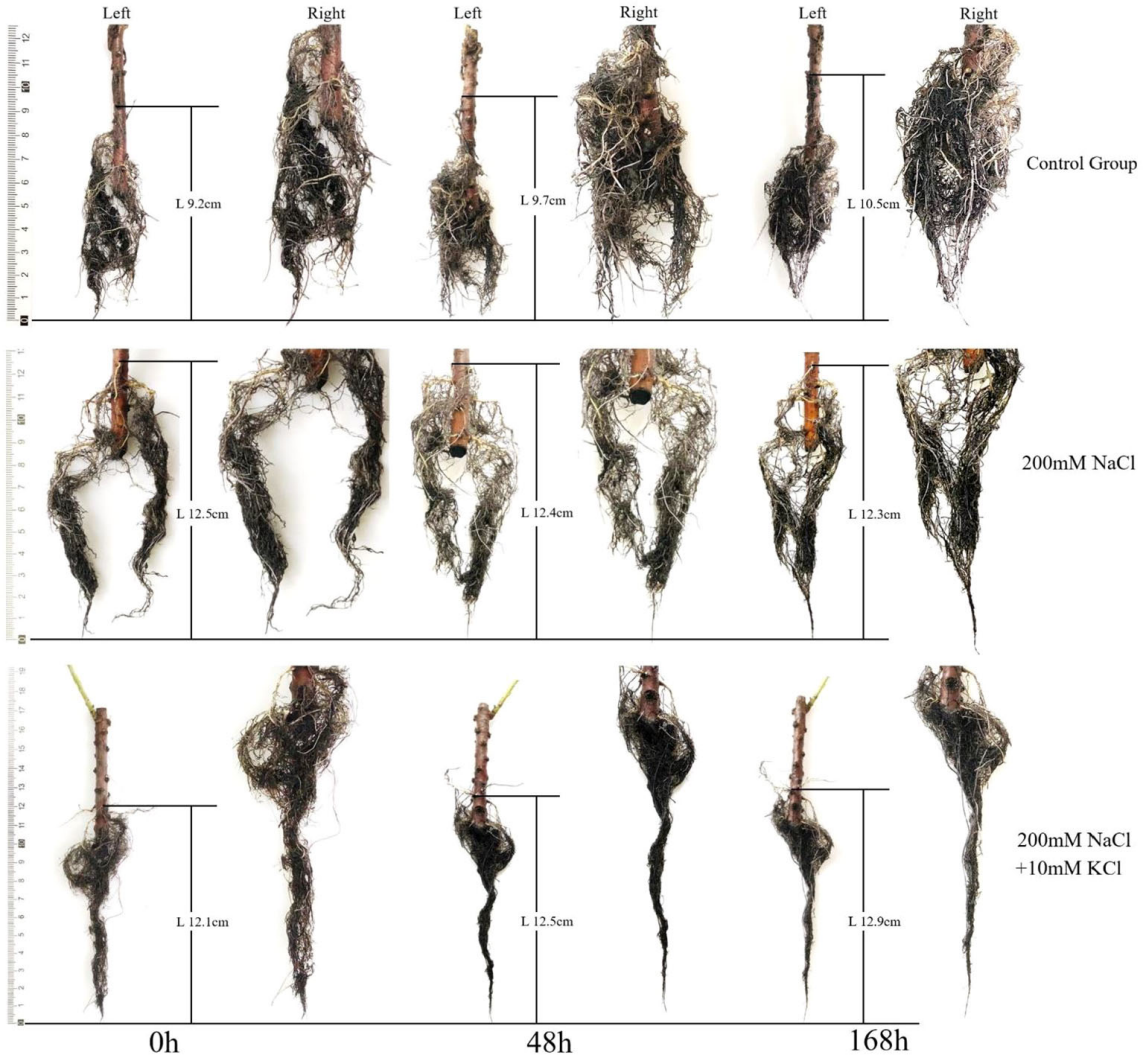
Change of roots growth of *T. ramosissima* under exogenous K^+^ application under NaCl stress. (Changes of the roots’ length of *T. ramosissima* in the control group and different treatments at 0h, 48h and 168h. The left is the original image, and the right is the zoomed image).

In this study, the length of the *T. ramosissima* roots increased and performed well in the control group over time. In the 200 mM NaCl group, the root lengths of *T. ramosissima* were slowly decreased at 48h and 168h with the increase of time. In the 200 mM NaCl + 10 mM KCl groups, the length of *T. ramosissima*’s roots has been growing slowly at 48h and 168h with the increase of time. The root length of the control group grew the most at 168h (**Figure 1**).

### 3.2 Antioxidant enzyme activity analysis of T. ramosissima roots by exogenous K^+^ application under NaCl stress

**Figure 2:**
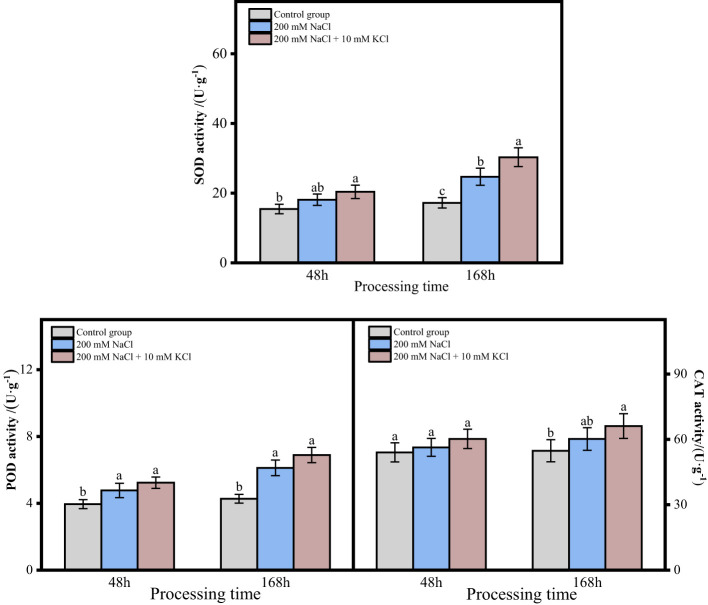
Change of antioxidative enzyme activity of *T. ramosissima* roots under NaCl stress with exogenous K^+^ application. (Changes of SOD, POD and CAT activities in the roots of *T. ramosissima* at 48h and 168h in the control group and different treatment groups. Different letters indicate the significance of differences among treatments at the same time, *p* < 0.05).

The activities of SOD, POD and CAT of roots of *T*. *ramosissima* in the 200 mM NaCl and 200 mM NaCl + 10 mM KCl groups showed an increasing trend with time when exogenous sources were applied under NaCl stress for 48 and 168 h (**Figure 2**). There was a significant increase in the activity of SOD in the 200 mM NaCl + 10 mM KCl group at 48h and 168h compared to the control group. The SOD activity of roots of *T*. *ramosissima* in the 200 mM NaCl group at 48h was increased compared to the control group, but not significantly. While the SOD activity of roots of *T*. *ramosissima* in the 200 mM NaCl group at 168h showed a significant increase compared to the control group. The POD activity of roots of *T*. *ramosissima* in the 200 mM NaCl group and 200 mM NaCl + 10 mM KCl group increased significantly at 48h and 168h compared to the control group. The CAT activity of roots of *T*. *ramosissima* in 200 mM NaCl group and 200 mM NaCl + 10 mM KCl group at 48h was slightly increased compared to control group and there was no significant difference. In contrast, the CAT activity of roots of *T*. *ramosissima* in the 200 mM NaCl + 10 mM KCl group at 168 h showed a significant increase compared to the control group.

As the research has demonstrated, the SOD, POD and CAT of *T*. *ramosissima* roots treated with 200 mM NaCl + 10 mM KCl were higher than those in the control group and 200 mM NaCl group within 168 h.

### 3.3 H_2_O_2_ and MDA content analysis of T. ramosissima roots by exogenous K^+^ application under NaCl stress

**Figure 3:**
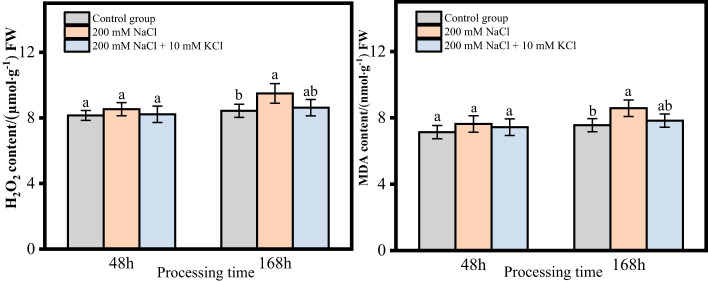
Change of H_2_O_2_ and MDA content of *T. ramosissima* roots under NaCl stress with exogenous K^+^ application (Changes of H_2_O_2_ and MDA content in the roots of *T. ramosissima* at 48h and 168h in the control group and different treatment groups. Different letters indicate the significance of differences among treatments at the same time, *p* < 0.05).

The contents of H_2_O_2_ and MDA in the roots of *T. ramosissima*, which treated with exogenous substances under NaCl stress, showed an increasing trend with time in both the 48h and 168h groups. In the 48h group, when exogenous K^+^ was applied under NaCl stress, the contents of H_2_O_2_ and MDA in the roots of *T. ramosissima*. slightly increased compared to the control group in both the 200 mM NaCl group and the 200 mM NaCl + 10 mM KCl group, but there was no significant change. In the 168h group, the contents of H_2_O_2_ and MDA in the roots of *T. ramosissima* significantly increased in the 200 mM NaCl group compared to the control group when exogenous K^+^ was applied under NaCl stress. However, the contents of H_2_O_2_ and MDA in the roots of *T. ramosissima* increased in the 200 mM NaCl + 10 mM KCl group compared to the control group, but the increase was not significant (**Figure 3**).

### 3.4 Analysis of differentially expressed genes related to SOD, POD and CAT activities at the roots of the T. ramosissima by exogenous K^+^ application under the NaCl stress

**Figure 4:**
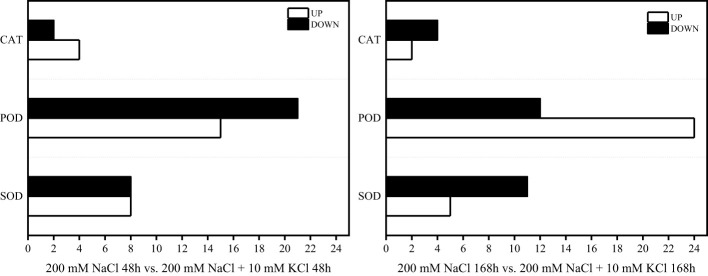
Statistical chart of the expression number of key genes of antioxidant enzymes in the roots of *T*. *ramosissima* with exogenous K^+^ applied under NaCl stress. (58 SOD, POD, CAT activities-related differences in the 200 mM NaCl 48h vs. 200 mM NaCl + 10 mM KCl 48h and 200 mM NaCl 168h vs. 200 mM NaCl + 10 mM KCl 168h comparison groups expressed genes were annotated to the KEGG pathway, and we counted the changes in the expression levels of these 58 DEGs in the 200 mM NaCl 48h vs. 200 mM NaCl + 10 mM KCl 48h and 200 mM NaCl 168h vs. 200 mM NaCl + 10 mM KCl 168h comparison groups).

According to the comparative analysis of transcription data of *T. ramosissima* treated with exogenous K^+^ for 0h, 48h and 168h under NaCl stress, significantly differential genes were screened with FDR < 0.05, corrected *p* < 0.05 and |log_2_FC| > 1. When *T. ramosissima* was exposed to exogenous K^+^ for 48h and 168h under NaCl stress, a total of 58 SOD, POD and CAT activity-related DEGs were altered in roots and annotated into the KEGG pathway. Among them, in the comparative analysis of 200 mM NaCl 48h vs. 200 mM NaCl + 10 mM KCl 48h, there were 27 up-regulated genes and 31 down-regulated genes; In the comparative analysis of 200 mM NaCl 168h vs. 200 mM NaCl + 10 mM KCl 168h, there were 31 up-regulated genes and 27 down-regulated genes. In the comparative analysis of 200 mM NaCl 48h vs. 200 mM NaCl + 10 mM KCl 48h, the highest number of up-regulated genes was POD (15), followed by SOD (8) and CAT (4); the highest number of down-regulated genes was POD (21), followed by SOD (8) and CAT (2). In the comparative analysis of 200 mM NaCl 168h vs. 200 mM NaCl + 10 mM KCl 168h, the highest number of up-regulated genes was POD (24), followed by SOD (5) and CAT (2); the highest number of down-regulated genes was POD (12), followed by SOD (11) and CAT (4) (**Figure 4**). The results showed that the number of up-regulated genes increased with the application of exogenous K^+^ under NaCl stress in *T. ramosissima*. Its antioxidant mechanism may initiate corresponding physiological responses to resist salt stress by up-regulating the expression of related genes. POD activity plays a major role during this period.

Under the stress of NaCl and with the treatment of exogenous K^+^ for 48h and 168h (**Table 1**), 4 DEGs including *Unigene0022482*, *Unigene0074194*, *Unigene0104234* and *Unigene0002762* in SOD activity, showed a trend of first decreasing and then increasing, and the expression level of *Unigene0035818* had been continuously increasing. 16 DEGs including *Unigene0002490*, *Unigene0004177*, *Unigene0016209, Unigene0022473*, *Unigene0033992*, *Unigene0009260*, *Unigene0034947*, *Unigene0053707*, *Unigene0068179*, *Unigene0076259*, *Unigene0080006*, *Unigene0084405*, *Unigene0084406*, *Unigene0087484*, *Unigene0089435* and *Unigene0102899* in POD activity showed a trend of first decreasing and then increasing. The expression levels of 8 DEGs such as *Unigene0013825*, *Unigene0014843*, *Unigene0021987*, *Unigene0029752*, *Unigene0033993*, *Unigene0052502*, *Unigene0053312* and *Unigene0090964* were continuously increasing. The expression levels of *Unigene0038031* and *Unigene0046159* DEGs in CAT activity were increasing. The results showed that the expression levels of these 31 DEGs were up-regulated within 168 h of exogenous K^+^ application under NaCl stress and were annotated to their associated KEGG pathway, and they play an important role in the resistance of *T. ramosissima* to salt stress.

**Table 1:** Analysis of DEGs with activity of major antioxidative enzymes.

Pathway	ID	Description	Log_2_ fold-change	
			200 mM NaCl 48h vs. 200 mM NaCl + 10 mM KCl 48h	200 mM NaCl 168h vs. 200 mM NaCl + 10 mM KCl 168h
SOD				
ko04146	*Unigene0022482*	SOD	-1.44	1.41
	*Unigene0033269*	SOD4 protein, partial	0.38	-0.75
	*Unigene0096238*	SOD	0.09	-1.88
	*Unigene0023980*	Iron/manganese superoxide dismutase	-7.46	-0.66
	*Unigene0027643*	Mn superoxide dismutase	-0.30	-0.27
	*Unigene0032758*	Superoxide dismutase, Fe-Mn family	-1.82	-4.29
	*Unigene0033135*	Manganese superoxide dismutase	2.47	-3.58
	*Unigene0034506*	Superoxide dismutase	0.12	-6.98
	*Unigene0035818*	Superoxide dismutase	8.61	2.08
	*Unigene0050462*	Superoxide dismutase	0.88	-0.07
	*Unigene0059078*	Cu Zn superoxide dismutase	7.91	-8.26
	*Unigene0074194*	Mitochondrial Mn superoxide dismutase	-1.10	4.91
	*Unigene0082550*	Superoxide dismutase	0.41	-0.09
	*Unigene0099972*	Superoxide dismutase [Cu-Zn]	-0.11	-2.10
	*Unigene0104234*	Superoxide dismutase [Cu-Zn] 1	-0.19	0.15
	*Unigene0002762*	Copper/zinc superoxide dismutase	-11.34	5.91
POD				
ko01100; ko01110; ko00940	*Unigene0000894*	Peroxidase 27-like	-0.15	-1.93
	*Unigene0002490*	Peroxidase 64-like	-0.51	1.26
	*Unigene0004177*	Peroxidase 3-like	-0.57	0.61
	*Unigene0009260*	Peroxidase 20	-1.30	2.05
	*Unigene0010026*	Peroxidase 29-like	1.47	-0.03
	*Unigene0013825*	Peroxidase	1.97	4.08
	*Unigene0013827*	Peroxidase	0.27	-0.32
	*Unigene0014843*	Peroxidase	1.79	1.81
	*Unigene0016209*	Peroxidase 27-like	-0.05	1.58
	*Unigene0021987*	Peroxidase 27-like	0.52	1.50
	*Unigene0022473*	Peroxidase A2-like	-0.16	1.07
	*Unigene0029752*	Peroxidase 17	0.34	0.75
	*Unigene0033992*	Peroxidase 3-like	-0.01	1.69
	*Unigene0033993*	Peroxidase 3-like	0.50	1.13
	*Unigene0034947*	Peroxidase 73-like	-0.38	0.71
	*Unigene0049353*	Peroxidase 5	2.53	-1.29
	*Unigene0052502*	Peroxidase 72	0.36	0.07
	*Unigene0053312*	Peroxidase 72-like	0.92	2.11
	*Unigene0053707*	Peroxidase 27	-0.43	0.79
	*Unigene0058419*	Peroxidase P7-like	0.94	-0.72
	*Unigene0064700*	Peroxidase 4	-1.47	-0.32
	*Unigene0068179*	Peroxidase 60-like	-1.29	0.21
	*Unigene0076135*	Peroxidase P7	0.66	-1.90
	*Unigene0076259*	Peroxidase 5-like	-0.19	0.99
	*Unigene0076940*	Peroxidase 60-like	1.49	-0.16
	*Unigene0079101*	Peroxidase P7	-0.47	-1.82
	*Unigene0080006*	Peroxidase 57-like	-0.22	2.45
	*Unigene0084405*	Peroxidase N-like	-0.14	0.99
	*Unigene0084406*	Peroxidase N	-0.10	2.17
	*Unigene0087484*	Peroxidase 11-like	-1.46	1.19
	*Unigene0089435*	Peroxidase 73-like	0.92	0.50
	*Unigene0090964*	Peroxidase	0.23	1.08
	*Unigene0092878*	Peroxidase 3-like	-1.18	-0.29
	*Unigene0094375*	Peroxidase 31	-0.93	-0.26
	*Unigene0102899*	Peroxidase 7	-0.83	0.50
	*Unigene0104832*	Peroxidase P7-like	-1.51	-0.53
CAT				
ko01100; ko01110; ko01200; ko00630; ko04146; ko04016; ko00380	*Unigene0038031*	Peroxisomal catalase-like	6.06	4.54
	*Unigene0046159*	Catalase isozyme 1	0.66	0.28
	*Unigene0046160*	Catalase, partial	-1.14	-1.49
	*Unigene0052968*	Catalase	0.26	-0.81
	*Unigene0068901*	Catalase	0.13	-5.52
	*Unigene0089761*	Catalase 3	-7.26	-0.57

### 3.5 Correlation analysis of transcriptomic and metabolomic data for major antioxidant enzyme activities (SOD, POD and CAT)

According to the requirements of the absolute value of the Person correlation coefficient, |Corr|>0.8, and *p*<0.05, the correlation analysis of 31 related DEGs obtained from SOD, POD and CAT activities in the transcriptome data and metabolome data was carried out.

The result indicates that 5 DEGs and 125 metabolites in the activity of SOD (**Supplementary Figure S1**), 24 DEGs and 139 metabolites in the activity of POD (**Supplementary Figure S2**), and 2 DEGs and 17 metabolites in the activity of CAT (**Supplementary Figure S3**) were in line with the absolute correlation coefficient of Person requirement for values |Corr|>0.8 and *p <*0.05. As a result, the DEGs in SOD, POD and CAT activities are mainly positively correlated with metabolites. It indicated that the primary antioxidant enzyme activity mechanism of *T*. *ramosissima* was dominated by the positive regulation of DEGs and their metabolites under NaCl stress for 48h and 168h. According to the metabolites screened by the 31 related DEGs in the above SOD, POD and CAT activities, it was found that 125 metabolites were significantly related to the 5 DEGs in the SOD activity, including 82 metabolites in the positive ion mode, and 19 metabolites were annotated to KEGG pathway; 43 metabolites in negative ion mode and 11 metabolites were annotated to KEGG pathways (**Supplementary Table S1**). There are 139 metabolites with a significant correlation with 24 DEGs in POD activity, of which 69 metabolites are in positive ion mode, and 15 metabolites are annotated to the KEGG pathway; there are 70 metabolites in negative ion mode, and 18 metabolites are annotated to KEGG pathway (**Supplementary Table S2**). There are 17 metabolites significantly correlated with 2 DEGs in CAT activity, of which 8 metabolites are in positive ion mode, and 2 metabolites are annotated to the KEGG pathway. Besides, there are 9 metabolites in negative ion mode (**Supplementary Table S3**).

As has been shown, most of the metabolites significantly correlated with the DEGs in SOD, POD and CAT activities are different, and their annotated KEGG pathways are also different.

### 3.6 Key KEGG pathway analysis of co-annotation of differentially expressed genes and differential metabolites

**Figure 5:**
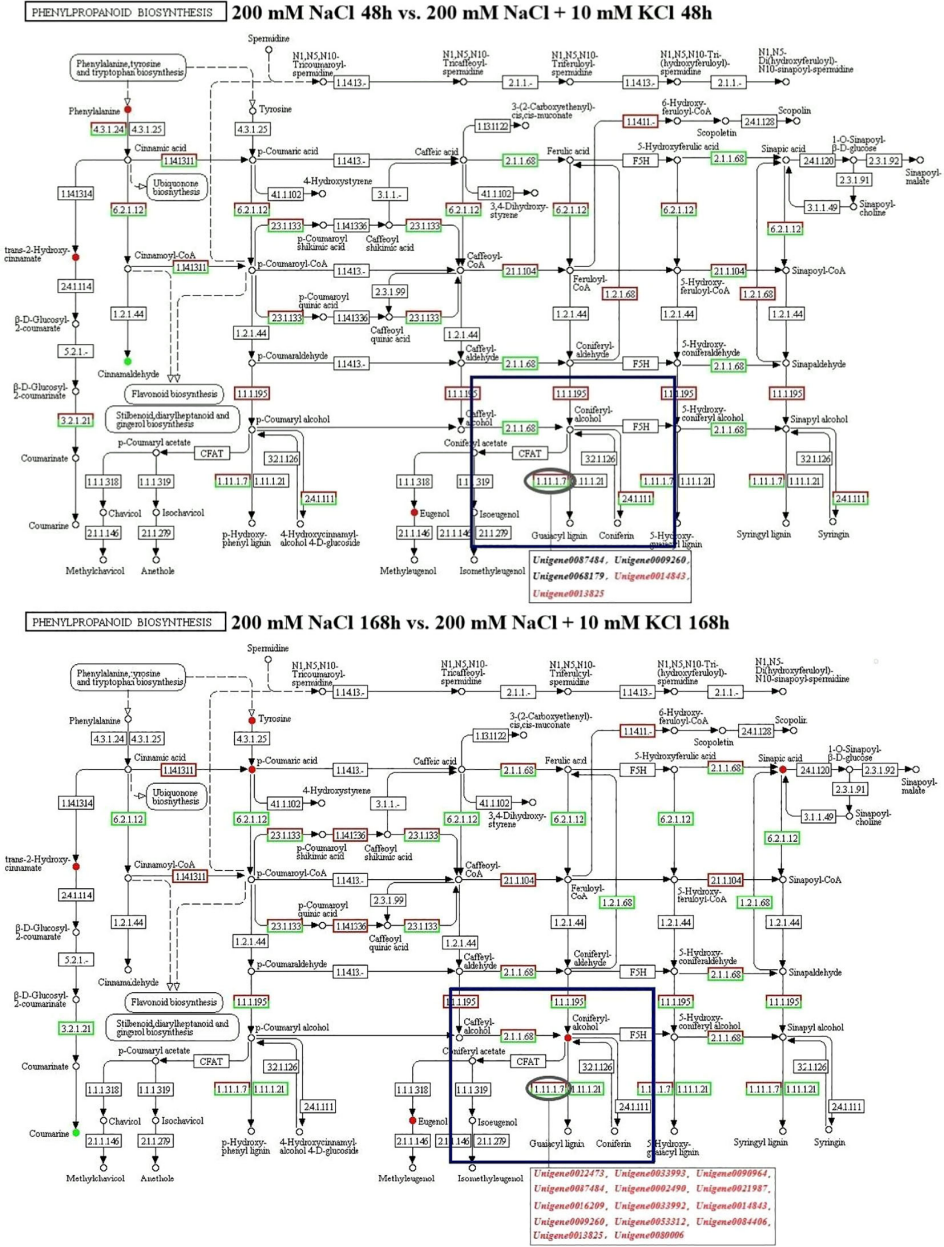
Analysis of Phenylpropanoid biosynthesis pathway in the roots of *T. ramosissima* by exogenous K^+^ application under NaCl stress. (1.11.1.7 in the blue box is the location of POD activity-related DEGs; these DEGs regulate the downstream of coniferyl alcohol; Black genes represent down-regulated genes, and red genes represent up-regulated genes).

**Figure 6:**
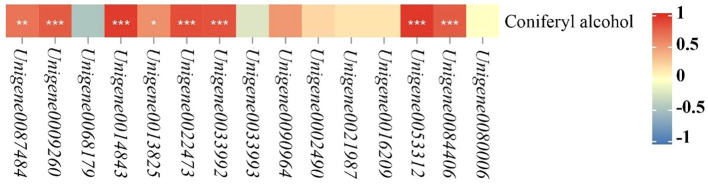
Heatmap analysis of the correlation between POD activity-related genes and Coniferyl alcohol. (In the phenylpropanoid biosynthesis pathway, heat map analysis of the correlation between 15 POD activity-related genes and coniferyl alcohol were generated. *p* ≥ 0.05 is not marked; 0.01 < *p* < 0.05 is marked as *0.001 < *p* < 0.01 is marked as **p ≤ 0.001 is marked as ***).

According to the KEGG pathway results of co-annotation of DEGs and differential metabolites, we found that only POD activity-related genes and their related metabolites were co-annotated to the phenylpropanoid biosynthesis pathway (**Figure 5**). In the phenylpropanoid biosynthesis pathway, there are 15 DEGs in POD activity including *Unigene0087484*, *Unigene0009260*, *Unigene0068179*, *Unigene0014843*, *Unigene0013825*, *Unigene0022473*, *Unigene0033993*, *Unigene0090964*, *Unigene0002490*, *Unigene0021987*, *Unigene0016209*, *Unigene0033992*, *Unigene0053312*, *Unigene0084406* and *Unigene0080006* were annotated to phenylpropanoid biosynthesis (ko00940) with coniferyl alcohol together, and these 15 DEGs regulate the downstream of coniferyl alcohol. In the 200 mM NaCl 48h vs. 200 mM NaCl + 10 mM KCl 48h comparison group, 3 DEGs, *Unigene0087484*, *Unigene0009260* and *Unigene0068179*, negatively regulated coniferyl alcohol, while both *Unigene0014843* and *Unigene0013825* DEGs positively regulated Coniferyl alcohol. In the 200 mM NaCl 168h vs. 200 mM NaCl + 10 mM KCl 168h comparison group, 14 DEGs including *Unigene0087484*, *Unigene0009260*, *Unigene0014843*, *Unigene0013825*, *Unigene0022473*, *Unigene0033993*, *Unigene0090964*, *Unigene0002490*, *Unigene0021987*, *Unigene0016209*, *Unigene0033992*, *Unigene0053312*, *Unigene0084406* and *Unigene0080006* positively regulated the downstream of coniferyl alcohol, which were positively correlated with coniferyl alcohol, and coniferyl alcohol appears to accumulate (**Supplementary Table S4**). Among them, *Unigene0087484*, *Unigene0009260*, *Unigene0014843*, *Unigene0013825*, *Unigene0022473*, *Unigene0033992*, *Unigene0053312* and *Unigene0084406* were significantly correlated with coniferyl alcohol (**Figure 6**). The results indicated that the transient expression of DEGs at the transcriptional level improved salt tolerance under NaCl stress for 48h in *T. ramosissima*. However, the number of DEGs related to POD activity increased with the increase of exogenous K^+^ addition time under NaCl stress. The positive regulation of related differential metabolites jointly resisted NaCl stress and alleviated NaCl damage.

### 3.7 Phylogenetic tree analysis of key candidate genes in POD activity

According to the association analysis of transcriptome and metabolome data in the phenylpropanoid biosynthesis pathway in 3.6, we found that *Unigene0014843* and *Unigene0013825* were in the phenylpropanoid biosynthesis pathway to positively regulate coniferyl alcohol in the POD activity of *T. ramosissima* and both *Unigene0014843* and *Unigene0013825* were significantly correlated with coniferyl alcohol. According to the expression levels of *Unigene0014843* and *Unigene0013825* at 200 mM NaCl and 200 mM NaCl + 10 mM KCl for 48h and 168h (**Supplementary Figure S4**), we observed that *Unigene0013825* responded significantly to the addition of exogenous K^+^ under NaCl stress. Its expression level first increased and then decreased in 200 mM NaCl treatment for 48h and 168h, and showed an upward trend in 200 mM NaCl + 10 mM KCl treatment for 48h and 168h. Therefore, we identified *Unigene0013825* as a key candidate gene in POD activity.

In this study, we selected the protein amino acid sequence of *Unigene0013825* to align on NCBI (National Center for Biotechnology Information) using BLAST and selected 15 homologous gene species (**Table 2**). The amino acid sequence of the *Unigene0013825* protein and the amino acid sequences of these 15 homologous gene species were used to construct a phylogenetic tree. The results showed that *Unigene0013825* was closely related to *Tamarix hispida* (**Supplementary Figure S5**).

**Table 2:** Information sheet for 15 species.

Family	Species	Description	Protein ID	CDS (bp)	ORF length (aa)
Malvaceae	*Theobroma cacao*	Predicted: peroxidase 66	XP_017973010.1	975	324
Amaranthaceae	*Spinacia oleracea*	Peroxidase 66-like	XP_021842095.1	972	323
Amaranthaceae	*Chenopodium quinoa*	Peroxidase 66-like	XP_021730481.1	972	323
Fagaceae	*Quercus suber*	Peroxidase 66-like	XP_023892517.1	1047	348
Malvaceae	*Corchorus olitorius*	Plant peroxidase	OMO97951.1	957	318
Rutaceae	*Citrus sinensis*	Peroxidase 66	XP_006490463.1	963	320
Anacardiaceae	*Pistacia vera*	Peroxidase 66-like	XP_031263699.1	972	323
Rosaceae	*Prunus dulcis*	Peroxidase 66	XP_034205436.1	975	324
Solanaceae	*Nicotiana tabacum*	Predicted: peroxidase 66-like	XP_016436625.1	972	323
Cannabaceae	*Cannabis sativa*	Peroxidase 66	XP_030500157.1	975	324
Moraceae	*Morus notabilis*	Peroxidase 66	XP_010106816.1	975	324
Oleaceae	*Olea europaea* var. *sylvestris*	Peroxidase 66	XP_022889445.1	966	321
Cleomaceae	*Tarenaya hassleriana*	Predicted: peroxidase 66	XP_010555640.1	966	321
Fabaceae	*Lupinus angustifolius*	Predicted: peroxidase 66	XP_019449837.1	972	323
Tamaricaceae	*Tamarix hispida*	Peroxidase	ACN60162.1	975	324

### 3.8 Quantitative real-time PCR validation of DEGs

**Figure 7:**
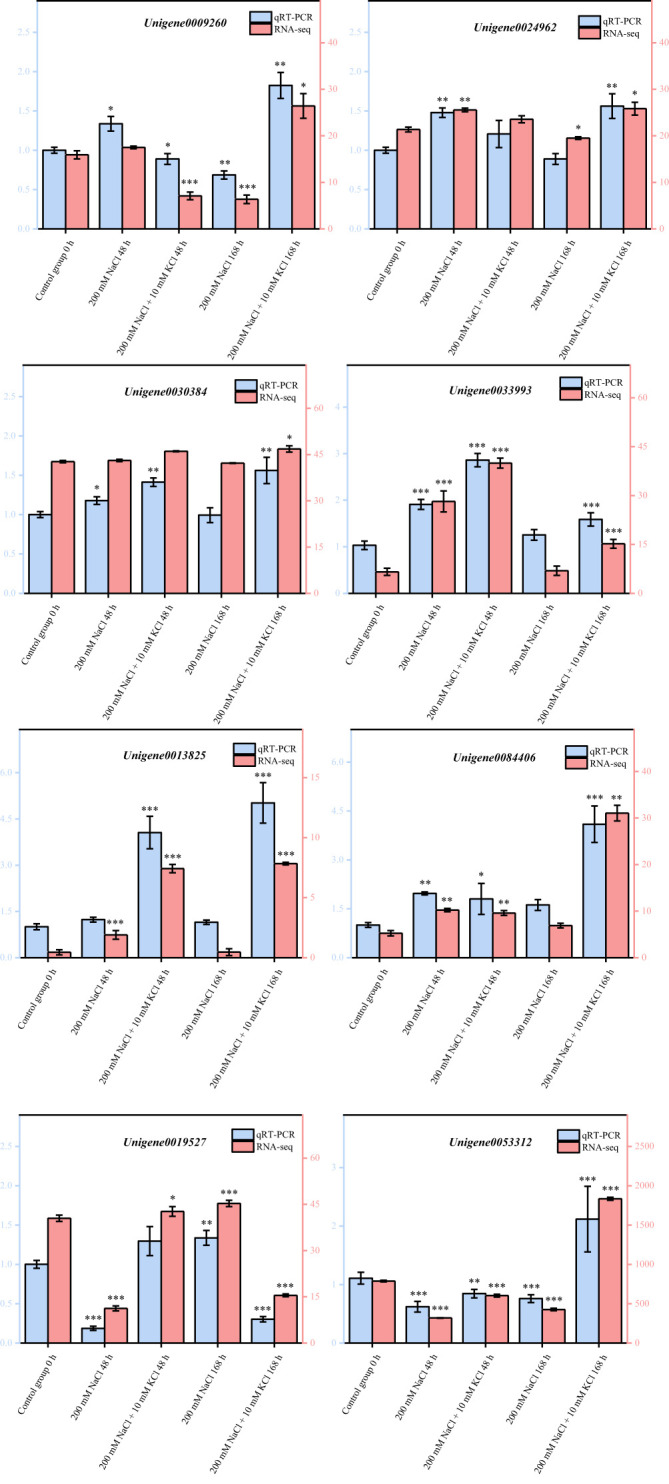
Validation of DEGs by qRT-PCR. (8 DEGs were randomly selected for quantitative real-time PCR (qRT-PCR) validation, the error bars were obtained from multiple replicates of qRT-PCR. Note: *p* ≥ 0.05 is not marked; 0.01 < *p* < 0.05 is marked as *0.001 < *p* < 0.01 is marked as **p ≤ 0.001 is marked as ***▬ qRT-PCR: Numerical value has been shown on the left side of the *Y* axis; ▬RNA-seq: Numerical value has been shown on the right side of the *Y* axis; all of the data inside the figure are relative quantitative values, no unit required).

The DEGs obtained *via* transcriptome sequencing were verified with qRT-PCR by selecting eight DEGs randomly in accordance with Chen et al. (2022b). In agreement with transcriptome sequencing analysis results (**Figure 7**), these qRT-PCR validation results were entirely consistent. As a result of this study, it has been demonstrated that transcriptome data obtained in this study are accurate and reliable, and can provide a theoretical basis for mining the genes related to K+ alleviation of NaCl stress and improving salt tolerance in *T*. *ramosissima*.

## 4 Discussion

Soil salinity is an important factor restricting global plant growth, and about 30% of the world’s arable land is adversely affected by the accumulation of salts (Epstein et al., 1980). Soil salinity is generally caused by excessive accumulation of NaCl, which adversely affects the environment where plant roots are located (Cakmak, 2005).

As a consequence of salt damage, ROS are produced, and the harmful cause of ROS is oxidative stress, which destroys the integrity of plant membranes and causes ion imbalance, generating ROS eventually, when excessively accumulated can cause oxidative stress, destroying biological macromolecules, biofilms, and other structures, leading to cell death in severe cases (Ahmad et al., 2010). Thus, salt-tolerant plants detoxify by enhancing their antioxidant defense systems (Zhu, 2000; Ali et al., 2011; Sun et al., 2011). In order to detoxify ROS, plants have developed antioxidant defences that include non-enzymatic antioxidants and enzymatic antioxidants, including SOD, POD, and CAT (Ali et al., 2011; Abbasi et al., 2014). Plant cells are primarily characterized by SOD, POD, and CAT enzymes that scavenge ROS. Under abiotic stress, the gene expression of these 3 types of enzymes increases along with the enhancement of enzyme activity, then the ability to scavenge ROS will also increase, thereby improving the resistance of plants to abiotic stress (Ali et al., 2011; Abbasi et al., 2014). The induction of ROS scavenging enzymes such as SOD, CAT, glutathione peroxidase (GPX), ascorbate peroxidase (APX), monodehydroascorbate reductase (MDHAR), dehydroascorbate reductase (DHAR), and glutathione transferases (GSTs) was positively correlated with the salt tolerance in plants (Sicilia et al., 2019). Studies have shown that under salt stress, the up-regulation of genes related to the activity of antioxidant enzymes such as SOD, POD and CAT can increase the antioxidant enzymes’ content and increase the ability to resist salt stress (Zhang et al., 2019). The expression level of the wheat *TaSOD1.7* gene was significantly increased in leaves, and its salt tolerance was enhanced (Jiang et al., 2019). The levels of DEGs related to SOD, POD, and CAT activities increased dramatically in leaves of *T. ramosissima* when exposed to NaCl stress, improving salt tolerance (Chen et al., 2022b). As a result of NaCl treatment for 48h and 168h, the activities of SOD, POD and CAT for the *T. ramosissima* roots were higher than those of the control group, and the expression levels of 58 DEGs related to these activities were altered. It has been shown that ROS produced by *T. ramosissima* under NaCl stress can result in damage to plants, but they can also activate plant antioxidant responses simultaneously and produce antioxidant enzymes (SOD, POD and CAT) to assist tissues in removing excess ROS in an orderly manner so that the redox balance of *T. ramosissima* tissue cells can be maintained, improving salt tolerance. However, the *Unigene0032758* gene was down-regulated in 200 mM NaCl 48h vs. 200 mM NaCl + 10 mM KCl 48h and 200 mM NaCl 168h vs. 200 mM NaCl + 10 mM KCl 168h comparison groups, similar to the results of Sicilia study (Sicilia et al., 2020). This indicates that the “neutralization” function of superoxide ions in the roots of *T. ramosissima* may be impaired, but it does not affect the normal growth of *T. ramosissima*.

K^+^ is the most abundant monovalent cation in higher plants and is essential for plant nutrition, growth, enzyme homeostasis and osmotic pressure regulation (Schachtman and Schroeder, 1994). In contrast to nitrogen and phosphorus, K^+^ does not participate in the composition of any organic matter in plants but plays an essential role in plant growth and development. By depolarizing K^+^ channels, Na^+^ competes for absorption through the root cytoplasmic membrane in saline soil, activating outwardly rectifying K^+^ channels. The ROS then activates K^+^ permeation channels in the roots, resulting in K^+^ efflux, reducing the K^+^/Na^+^ ratio, and ultimately producing Na^+^ toxicity in plants. Previous studies have shown that improving plants’ K^+^ nutritional status can greatly reduce ROS production (Ahmad et al., 2010). For example, applying K^+^ humate increased SOD, POD and CAT activities and delayed roots senescence (Liang et al., 2007). In addition, applying KNO_3_ can alleviate the salinity effect of winter wheat by increasing antioxidant enzyme activity (Zheng et al., 2008). Finally, the K^+^ application in plants under salt stress can enhance the scavenging ability of the scavenging system for ROS (Abbasi et al., 2016).

Furthermore, plants consuming K^+^ in environments with high NaCl or low K^+^ concentrations can enhance the root length and fresh weight and enhance salt tolerance (Ardie et al., 2009; Ren et al., 2015). In this study the control group had the most increase in root length and better root growth, followed by the 200 mM NaCl + 10 mM KCl group with increased root length, but the 200 mM NaCl group’s root length was decreased. This suggested that K^+^ can significantly improve plant metabolism in salt environments. The roots of *T*. *ramosissima* maintain the balance of the K^+^/Na^+^ ratio by absorbing K^+^ and resisting salt stress. The content of H_2_O_2_ and MDA in the roots of *T. ramosissima* were increased in all treatment groups, and 200 mM NaCl group increased the most. The roots of *T. ramosissima* were exposed to exogenous K^+^ for 48h and 168h under NaCl stress, and the activities of SOD, POD and CAT were significantly increased. The SOD, POD, and CAT activities in the 200 mM NaCl + 10 mM KCl group were higher than those in the 200 mM NaCl group. Particularly, the roots of *T. ramosissima* were exposed to exogenous K^+^ for 168h under NaCl stress, and the activities of SOD, POD and CAT were significantly higher than those of the control group. The number of up-regulated DEGs related to SOD, POD and CAT increased. Notably, the DEGs related to POD activity increased the most, which played an important role in enhancing the salt tolerance of *T. ramosissima* and alleviating NaCl toxicity.

Additionally, the products of the phenylpropane metabolic pathway play a certain role in the process of scavenging ROS. 3 hydroxycinnamyl alcohol precursors in the phenylpropanoid biosynthesis pathway, namely p-coumaroyl, coniferyl and sinapyl alcohol, derive p-hydroxybenzyl (H-lignin), guaiacyl (G-lignin) and syringyl lignin (S-lignin), respectively (Chen et al., 2020). Lignin in plants affects plant tolerance to abiotic stress (Li et al., 2020). POD is an enzyme widely present in plant tissues and exists in various molecular forms. It has been used as a biochemical marker for various biotic and abiotic stresses (Penel et al., 1992). Its involvement in wound and salt stress responses may be related to its catalytic role in cell wall pectin and structural protein cross-linking. Coniferyl alcohol is an organic compound used as a substrate marker for peroxidases that may be involved in lignification. It has been reported that the peroxidase in plant roots has the highest catalytic efficiency when coniferyl alcohol is used as the substrate (Quiroga et al., 2001). In this study, 5 POD-related DEGs were found to regulate the downstream of coniferyl alcohol in the phenylpropanoid biosynthesis pathway under NaCl stress for 48h. Among them, 3 DEGs, including *Unigene0087484*, *Unigene0009260* and *Unigene0068179*, negatively regulate coniferyl alcohol, while *Unigene0014843* and *Unigene0013825* positively regulate coniferyl alcohol, but there was no significant difference in coniferyl alcohol. When exogenous K^+^ was applied for 168 h under NaCl stress, it was found that 14 DEGs such as *Unigene0087484*, *Unigene0009260*, *Unigene0014843*, *Unigene0013825*, *Unigene0022473*, *Unigene0033993*, *Unigene0090964*, *Unigene0002490*, *Unigene0021987*, *Unigene0016209*, *Unigene0033992*, *Unigene0053312*, *Unigene0084406* and *Unigene0080006* positively regulated the downstream of coniferyl alcohol, which was positively correlated with coniferyl alcohol, and there were significant differences in coniferyl alcohol. The results indicated ROS production occurred during applying exogenous K^+^ in *T. ramosissima* under NaCl stress. The deleterious effects of these ROS were alleviated by enhancing POD activity. These findings are also consistent with the research idea that the antioxidant activity of plants under NaCl stress is enhanced by K^+^ application (Zheng et al., 2008; Abbasi et al., 2014). Simultaneously, it was observed in this study that the expression level of *Unigene0013825* with POD activity initially increased and then decreased under NaCl stress for 48h and 168h, respectively, while the exogenous K^+^ expression level increased under NaCl stress. The results indicate that *Unigene0013825* is essential in enhancing POD activity and is the key differentially expressed gene that enhances POD activity. Exogenous K^+^ can be given to *T. ramosissima* when subjected to NaCl stress in order to increase antioxidant enzyme activity and strengthen resistance to salt stress.

## 5 Conclusion

Under the influence of exogenous K^+^ addition under NaCl stress, the root of *T. ramosissima* increased its antioxidant enzyme activity, which enhanced the ability of the *T. ramosissima* to resist NaCl stress and maintained the normal growth of *T. ramosissima*. The application of exogenous K^+^ under NaCl stress played an important role in alleviating NaCl toxicity by the antioxidant enzyme activity system in the roots of *T. ramosissima*. This study provides genetic resources and a scientific theoretical basis for further breeding of salt-tolerant *Tamarix* plants and the molecular mechanism of K^+^ alleviating NaCl toxicity.

## Data availability statement

The datasets presented in this study can be found in online repositories. The names of the repository/repositories and accession number(s) can be found in the article/**Supplementary Material.**

## Author contributions

YC, JZ, ZS and JJ contributed to conception and design of the study. YC, HL, SD and SZ organized the database. YC and SZ performed the statistical analysis. YC wrote the first draft of the manuscript. YC wrote sections of the manuscript. All authors contributed to the article and approved the submitted version.
