# Supplementary material for: Analysis of the main antioxidant enzymes in the roots of Tamarix ramosissima under NaCl stress by applying exogenous potassium (K+)
Source: Front Plant Sci. 2023 Apr 18;14:1114266. doi: 10.3389/fpls.2023.1114266 (PMC10151674; doi:10.3389/fpls.2023.1114266)
Supplement: Supplementary file 1 [file DataSheet_1.pdf]

Supplementary Table S1. Key differential metabolites Log_2 fold-change in SOD activity

Name	Formula	RT [min]	m/z	Control group-0h		200 mM NaCl-48h		200 mM NaCl + 10 mM KCl-48h		200 mM NaCl-168h		200 mM NaCl + 10 mM KCl-168h		Pathway
				Mean	Standard deviation	Mean	Standard deviation	Mean	Standard deviation	Mean	Standard deviation	Mean	Standard deviation	
**Positive**														
DGTS (2:0/16:4)	$C_{28}H_{45}NO_7$	12.50	508.33	16.80	0.33	20.78	0.35	19.01	0.53	16.55	0.20	17.28	1.00	
(-)-Caryophyllene oxide	$C_{15}H_{24}O$	14.72	221.19	26.38	0.04	26.17	0.16	26.18	0.04	26.40	0.06	26.03	0.02	
(-)-Epigallocatechin	$C_{15}H_{14}O_7$	6.10	289.07	22.92	0.92	25.69	0.31	23.04	0.21	24.23	0.10	23.93	0.08	ko01110; ko00941
Methyl dihydrojasmonate	$C_{13}H_{22}O_3$	13.28	227.16	23.13	0.07	22.82	0.12	24.21	0.21	24.22	0.07	24.27	0.05	
Oryzalexin E	$C_{20}H_{32}O_2$	15.06	305.25	22.33	0.16	23.28	0.32	21.90	0.22	23.63	0.28	22.08	0.12	ko01110
5α-Dihydrotestosterone glucuronide	$C_{25}H_{38}O_8$	12.31	467.26	17.43	0.21	23.70	0.28	20.27	0.43	16.81	0.02	18.69	0.18	
DGTS (5:0/13:1)	$C_{28}H_{51}NO_7$	13.20	514.37	21.64	0.31	23.20	0.50	23.11	0.41	21.98	0.12	21.48	0.15	
PC (2:0/16:3)	$C_{26}H_{46}NO_8P$	12.61	532.30	18.71	0.24	21.11	0.92	20.15	0.38	19.70	0.13	18.96	0.45	
LDGTS 16:2	$C_{26}H_{47}NO_6$	13.93	470.35	21.05	0.41	24.60	0.25	20.41	0.40	20.55	0.19	20.09	0.23	
4-Methoxybenzaldehyde	$C_8H_8O_2$	5.90	137.06	23.68	0.40	24.81	0.15	23.76	0.10	23.79	0.10	22.89	0.29	
LDGTS 18:1	$C_{28}H_{53}NO_6$	14.69	500.39	23.38	0.35	20.73	0.89	18.95	0.14	19.01	0.02	18.94	0.05	
Scopolin	$C_{16}H_{18}O_9$	7.69	355.10	23.18	0.33	23.17	0.17	21.24	0.50	23.42	0.06	21.44	0.20	ko01110; ko00940
O-7460	$C_{25}H_{48}FO_5P$	14.15	496.36	23.65	0.26	26.66	0.16	23.45	0.41	22.56	0.39	22.65	0.40	
2,3-Dinor-8-epi-prostaglandin F2α	$C_{18}H_{30}O_5$	12.64	327.22	20.97	0.07	23.18	0.14	21.89	0.16	23.36	0.31	22.26	0.18	
3,4-MDEA-d5	$C_{12}H_{12}H_5NO_2$	13.07	213.16	18.05	0.39	21.46	0.14	19.29	0.12	18.69	0.09	18.81	0.15	
15-Deoxy-Δ12,14-prostaglandin A1	$C_{20}H_{30}O_3$	14.38	301.22	20.67	0.32	19.67	0.12	19.80	0.29	21.75	0.20	20.50	0.38	
p-Coumaric acid	$C_9H_8O_3$	6.26	165.05	24.52	0.24	23.68	0.19	24.61	0.15	23.91	0.06	24.82	0.21	ko01100; ko01110; ko00940; ko00360; ko00950; ko01220; ko00350; ko00130
PC (18:5e/2:0)	$C_{28}H_{48}NO_7P$	14.11	542.32	23.40	0.30	23.86	0.31	22.81	0.16	22.96	0.07	23.09	0.95	
4-methyl-5-oxo-2-pentyl-2,5-	$C_{11}H_{16}O_4$	11.17	213.11	19.97	0.03	20.19	0.12	20.12	0.09	20.13	0.28	20.45	0.10	

dihydrofuran-3-carboxylic acid														
Milbemycin A4 oxime	$C_{32}H_{45}NO_7$	12.69	538.32	20.14	0.05	22.96	0.38	21.25	0.02	20.66	0.24	20.07	0.10	
Acetyl-L-carnitine	$C_9H_{17}NO_4$	1.76	204.12	23.26	0.04	22.47	0.13	23.33	0.12	24.64	0.21	23.43	0.23	ko04931
N-Acetyl-L-valine	$C_7H_{13}NO_3$	1.32	160.10	24.96	0.27	23.48	0.06	23.65	0.26	24.23	0.11	23.71	0.01	
19-Nortestosterone	$C_{18}H_{26}O_2$	13.02	275.20	23.75	0.14	26.71	0.02	24.45	0.09	23.99	0.06	24.29	0.08	
Kahweol	$C_{20}H_{26}O_3$	12.99	315.19	20.83	0.48	20.41	0.30	19.53	0.25	20.52	0.31	19.94	0.20	
LDGTS 16:4	$C_{26}H_{43}NO_6$	13.39	466.32	19.29	0.19	22.27	0.19	17.63	1.74	17.55	1.50	16.08	0.06	
Taxifolin	$C_{15}H_{12}O_7$	7.88	305.06	20.42	0.34	22.58	0.23	21.62	0.38	21.41	0.12	21.03	0.19	ko01100; ko01110; ko00941
SQDG (18:2/16:4)	$C_{43}H_{70}O_{12}S$	13.98	828.51	19.25	0.04	22.29	0.36	18.70	0.08	18.97	0.07	18.88	0.05	
LPC 16:2	$C_{24}H_{46}NO_7P$	13.96	492.31	22.43	0.19	24.44	0.12	23.25	0.25	23.31	1.23	23.13	1.00	
Nicotinamide	$C_6H_6N_2O$	1.96	123.06	25.51	0.13	27.68	0.19	26.00	0.20	27.49	0.10	25.77	0.38	ko01100; ko00760
Alisol B	$C_{30}H_{48}O_4$	14.80	495.35	25.76	0.19	23.91	0.13	24.59	0.48	24.12	0.11	24.53	0.14	
Thymol	$C_{10}H_{14}O$	10.99	151.11	21.42	0.05	21.51	0.16	21.41	0.08	21.66	0.13	21.12	0.08	
C-8 Ceramide-1-phosphate	$C_{26}H_{52}NO_6P$	12.19	528.35	18.73	0.15	20.46	0.73	20.38	0.47	18.14	0.13	18.71	0.32	
Estriol	$C_{18}H_{24}O_3$	12.77	289.18	19.86	0.18	22.59	0.07	20.54	0.11	20.19	0.14	19.81	0.17	ko01100
Palmitoleic Acid	$C_{16}H_{30}O_2$	14.60	277.22	21.81	0.14	22.48	0.24	22.01	0.08	22.38	0.20	21.91	0.07	ko00061
7α-Hydroxytestosterone	$C_{19}H_{28}O_3$	13.44	305.21	20.80	0.20	23.73	0.01	21.89	0.32	20.73	0.07	20.46	0.12	
Edpetiline	$C_{33}H_{53}NO_8$	13.88	592.39	19.01	0.30	24.41	0.27	18.82	0.17	19.96	0.60	18.94	0.31	
2,3-Dinor-11β-prostaglandin F2α	$C_{18}H_{30}O_5$	12.48	349.20	19.61	0.12	20.80	0.22	20.87	0.06	22.58	0.10	20.65	1.14	
Isotretinoin	$C_{20}H_{28}O_2$	14.65	301.22	22.83	0.22	23.21	0.32	21.56	0.17	24.43	0.15	22.57	0.17	
4-Fluoro-α-pyrrolidinobutiophenone	$C_{14}H_{18}FNO$	1.36	236.15	25.09	0.17	26.87	0.12	23.92	0.32	23.96	0.10	23.47	0.23	
8,8-dimethyl-2-phenyl-4H,8H-pyrano[2,3-h]chromen-4-one	$C_{20}H_{16}O_3$	14.37	305.12	19.53	0.22	22.73	0.37	19.11	0.44	20.28	0.02	19.81	0.12	
Amitriptyline-d3	$C_{20}H_{20}H_3N$	13.37	281.21	18.92	0.14	20.69	0.33	19.70	0.11	18.85	0.14	18.49	0.19	
Diaminopimelic acid	$C_7H_{14}N_2O_4$	1.34	174.08	24.09	0.05	22.73	0.18	23.34	0.06	24.07	0.09	23.05	0.22	ko01100; ko01110; ko01120; ko01130; ko01230; ko00300
Kynurenic acid	$C_{10}H_7NO_3$	7.66	190.05	25.29	0.11	19.38	0.25	21.10	0.36	21.60	0.29	21.29	0.46	ko01100;

														ko00380
LPC 18:4	$C_{26}H_{46}NO_7P$	13.86	516.31	19.92	0.41	22.02	0.66	20.12	0.32	19.28	0.23	18.97	0.02	
Glycocholic acid	$C_{26}H_{43}NO_6$	12.27	466.32	20.41	0.13	19.59	0.42	23.14	0.28	21.65	0.05	22.14	0.07	ko01100
LPC 20:3	$C_{28}H_{52}NO_7P$	11.13	546.36	17.58	0.09	19.49	0.30	19.16	0.36	17.82	0.04	17.54	0.18	
10-Nitrolinoleate	$C_{18}H_{31}NO_4$	13.30	343.26	21.66	0.18	21.13	0.19	21.92	0.21	20.19	0.09	20.83	0.16	
Daunorubicin	$C_{27}H_{29}NO_{10}$	9.05	528.18	25.20	0.06	22.55	1.02	24.17	0.12	23.22	0.21	23.20	0.10	ko01130
Tiglic acid	$C_5H_8O_2$	1.43	101.06	26.56	0.35	25.69	0.42	26.67	0.08	27.22	0.05	26.03	0.06	
2-[5-(2-hydroxypropyl)oxolan-2-yl]propanoic acid	$C_{10}H_{18}O_4$	12.31	185.12	21.92	0.05	21.34	0.17	21.41	0.64	21.94	0.84	21.77	0.21	
Biotin	$C_{10}H_{16}N_2O_3S$	9.14	245.09	29.00	0.07	27.77	0.19	28.05	0.08	27.79	0.15	28.10	0.04	ko01100; ko02010; ko00780
PC (18:3e/2:0)	$C_{28}H_{52}NO_7P$	14.62	546.36	23.17	0.12	22.56	0.14	20.38	0.44	21.34	0.24	21.91	0.86	
LysoPC 18:0	$C_{26}H_{54}NO_7P$	14.59	546.35	23.12	0.12	22.58	0.07	20.31	0.46	21.23	0.38	21.87	0.85	
Carvone	$C_{10}H_{14}O$	13.26	151.11	20.78	0.09	21.58	0.11	21.74	0.02	21.42	0.17	21.14	0.05	ko01110; ko00902; ko00903
Isorhamnetin	$C_{16}H_{12}O_7$	9.53	317.07	22.21	0.47	26.88	0.15	25.94	0.27	26.34	0.19	26.87	0.70	
Echinocystic acid	$C_{30}H_{48}O_4$	13.00	237.18	22.97	0.55	21.90	0.35	21.84	0.13	22.34	0.27	21.41	0.04	
1-[4-hydroxy-3-(3-methylbut-2-en-1-yl)phenyl]ethan-1-one	$C_{13}H_{16}O_2$	11.83	205.12	20.81	0.17	20.90	0.06	20.69	0.08	21.08	0.10	20.83	0.09	
15-Acetyldeoxynivalenol	$C_{17}H_{22}O_7$	10.38	321.13	21.08	0.17	22.35	0.16	21.10	0.10	22.86	0.04	21.17	0.15	
17α-Ethynylestradiol	$C_{20}H_{24}O_2$	12.24	297.18	20.68	0.75	19.65	0.08	20.19	0.08	20.74	0.32	20.21	0.87	
YLK	$C_{21}H_{34}N_4O_5$	14.22	405.25	24.82	0.21	18.74	0.06	22.65	0.25	22.93	0.24	22.68	0.05	
Cafestol	$C_{20}H_{28}O_3$	13.61	317.21	22.29	2.07	23.97	0.49	24.76	0.33	24.82	0.26	25.19	0.09	
N-cyclohexyl-1-methyl-5-(1H-pyrrol-1-yl)-1H-pyrazole-4-carboxamide	$C_{15}H_{20}N_4O$	14.37	291.18	19.33	0.26	22.00	0.18	19.22	0.09	19.79	0.08	19.24	0.30	
Testosterone	$C_{19}H_{28}O_2$	14.08	289.22	19.20	0.58	20.34	0.44	19.01	0.43	19.13	0.21	18.82	0.16	ko01100; ko01120
7-(2-hydroxypropan-2-yl)-1,4a-dimethyl-decahydronaphthalen-1-ol	$C_{15}H_{28}O_2$	13.47	223.21	22.54	0.08	20.77	0.50	20.88	0.18	23.64	0.30	19.94	0.16	
Prostaglandin K2	$C_{20}H_{30}O_5$	12.82	333.20	25.14	0.11	25.81	0.45	25.50	0.15	26.66	0.14	25.42	0.09	
Isorhapontigenin	$C_{15}H_{14}O_4$	9.37	259.10	26.81	0.08	28.91	0.09	28.01	0.32	27.92	0.04	27.38	0.32	

Eicosapentaenoic acid	$C_{20}H_{30}O_2$	13.94	303.23	23.84	0.05	24.05	0.20	23.94	0.31	25.00	0.32	23.38	0.43	ko01040
LDGTS 18:4	$C_{28}H_{47}NO_6$	13.87	239.16	22.02	0.14	24.60	0.30	22.19	0.45	22.21	1.17	21.91	0.51	
DGTS (2:0/16:3)	$C_{28}H_{47}NO_7$	12.83	510.34	19.42	0.64	23.21	0.37	22.17	0.18	20.27	0.54	20.46	0.71	
Phloretin	$C_{15}H_{14}O_5$	7.68	275.09	24.74	0.08	27.03	0.16	25.67	0.22	25.95	0.21	24.86	0.07	ko01110; ko00941
4-Methoxycinnamic acid	$C_{10}H_{10}O_3$	14.64	179.07	27.84	0.08	19.67	0.01	26.49	0.19	24.50	0.14	26.11	0.02	
PC (2:0/16:2)	$C_{26}H_{48}NO_8P$	12.96	534.32	23.33	0.17	24.41	0.26	24.65	0.05	22.50	0.60	21.89	0.32	
LDGTS 16:1	$C_{26}H_{49}NO_6$	14.25	472.36	23.80	0.25	24.07	0.32	22.40	0.62	23.05	0.32	21.62	1.20	
SM (d14:2/12:1)	$C_{31}H_{59}N_2O_6P$	13.63	587.43	18.51	0.12	18.81	0.16	19.69	1.20	18.34	0.07	20.43	0.11	
MQH	$C_{16}H_{26}N_6O_5S$	12.98	415.17	19.10	0.14	19.46	0.53	18.49	0.18	19.43	1.19	18.67	0.09	
4-Pyridoxic acid	$C_8H_9NO_4$	1.28	184.06	23.42	0.61	22.60	0.15	20.48	0.25	23.29	0.14	20.84	0.13	ko01100; ko01120; ko00750
Hirsuteine	$C_{22}H_{26}N_2O_3$	12.91	367.21	20.78	0.02	24.18	0.15	22.27	0.46	22.27	0.06	21.64	0.04	
1-Palmitoylglycerol	$C_{19}H_{38}O_4$	14.45	331.28	25.48	0.06	26.16	0.12	25.55	0.59	25.82	0.07	25.27	0.07	
LDGTS 16:3	$C_{26}H_{45}NO_6$	13.61	468.33	19.19	0.25	24.01	0.16	20.25	1.05	19.09	0.33	19.55	0.56	
3,4-Methylenedioxy-α-pyrrolidinopropiophenone	$C_{14}H_{17}NO_3$	7.15	248.13	25.62	0.05	23.52	0.04	25.20	0.06	23.36	0.07	23.79	0.07	
LPE 18:4	$C_{23}H_{40}NO_7P$	12.97	474.26	19.30	0.27	21.80	0.80	21.53	0.36	19.95	0.30	20.84	0.22	
13,14-dihydro-15-keto-tetranor Prostaglandin E2	$C_{16}H_{26}O_5$	12.73	281.17	21.15	0.03	21.39	0.07	20.99	0.16	21.26	0.56	20.81	0.10	
**Negative**														
PE (2:0/16:3)	$C_{23}H_{40}NO_8P$	12.80	488.24	16.78	0.46	19.76	0.51	20.17	0.19	16.56	0.15	18.24	0.11	
Sinigrin	$C_{10}H_{17}NO_9S_2$	8.84	358.03	23.95	0.12	25.22	0.04	21.66	0.37	24.36	0.08	24.26	0.25	
PA (2:0/20:4)	$C_{25}H_{41}O_8P$	12.12	499.26	19.63	0.29	25.36	0.28	22.50	0.47	19.04	0.09	20.84	0.04	
DGDG (16:1/18:5)	$C_{49}H_{80}O_{15}$	14.27	967.55	17.50	0.47	24.43	0.01	20.13	0.48	20.44	0.42	20.69	0.13	
Sclareolide	$C_{16}H_{26}O_2$	12.79	249.19	20.82	0.12	20.31	0.16	20.71	0.23	20.56	0.03	20.90	0.11	
(+/-)11(12)-EET	$C_{20}H_{32}O_3$	13.35	319.23	23.25	0.20	22.87	0.23	22.82	0.61	23.69	0.09	22.38	0.16	
Ingenol-5,20-acetonide	$C_{23}H_{32}O_5$	13.62	387.22	20.88	0.16	20.04	0.09	20.53	0.08	22.02	0.08	21.28	0.06	
LPS 17:2	$C_{23}H_{42}NO_9P$	12.98	506.25	18.41	0.50	20.89	0.52	21.39	0.12	20.30	0.76	20.80	0.13	
D-(-)-Mannitol	$C_6H_{14}O_6$	1.30	181.07	24.97	0.32	25.78	0.10	24.93	0.05	26.62	0.09	25.34	0.12	
PE (2:0/16:2)	$C_{23}H_{42}NO_8P$	12.97	490.26	20.93	1.53	20.54	0.23	21.05	0.27	19.36	0.03	20.34	0.18	
14,15-Dehydrocrepenynic acid	$C_{18}H_{28}O_2$	13.97	275.20	23.05	0.33	24.19	0.20	22.95	0.13	23.51	0.69	22.61	0.11	

MAG (18:3)	$C_{21}H_{36}O_4$	13.88	351.25	23.61	0.18	23.47	0.23	21.59	0.30	23.26	0.01	21.85	0.11	
20-Hydroxy-(5Z,8Z,11Z,14Z)-eicosatetraenoic acid	$C_{20}H_{32}O_3$	13.21	319.23	21.45	0.21	20.71	0.42	21.06	0.12	23.19	0.05	21.79	0.13	ko01100; ko00590
(+/-)18-HEPE	$C_{20}H_{30}O_3$	13.02	317.21	21.37	0.30	24.30	0.22	21.52	0.15	24.22	0.14	22.80	0.09	
Arachidonic acid	$C_{20}H_{32}O_2$	14.47	303.23	27.56	0.29	27.73	0.41	26.20	0.18	28.26	0.08	27.04	0.19	ko01100; ko01040; ko00590; ko00591
PE (22:4e/3:0)	$C_{30}H_{54}NO_7P$	13.05	570.37	17.87	0.40	21.12	0.47	20.29	0.29	17.79	0.03	19.57	0.37	
16-Hydroxyhexadecanoic acid	$C_{16}H_{32}O_3$	14.33	253.22	31.48	0.19	29.87	0.32	30.23	0.20	30.70	0.10	30.14	0.18	
cis-5,8,11,14,17-Eicosapentaenoic acid	$C_{20}H_{30}O_2$	14.20	301.22	25.74	0.38	28.70	0.43	25.81	0.20	28.40	0.13	27.46	0.15	ko01040
13(S)-HOTrE	$C_{18}H_{30}O_3$	13.25	293.21	27.70	0.16	26.39	0.18	27.97	0.24	27.63	0.07	28.43	0.12	ko00592
Sucralose	$C_{12}H_{19}Cl_3O_8$	9.80	395.01	27.25	0.31	23.27	0.07	22.64	0.34	25.12	0.09	23.61	0.65	
13,14-dihydro-15-keto-tetranor Prostaglandin D2	$C_{16}H_{26}O_5$	11.38	279.16	18.17	0.18	20.73	0.13	19.75	0.27	18.63	0.09	18.75	0.18	
9,17-Octadecadiene-12,14-diyne-1,11,16-triol	$C_{18}H_{26}O_3$	12.75	289.18	17.35	0.25	18.51	0.52	17.44	0.25	17.57	0.08	17.39	0.11	
5'-S-Methyl-5'-thioadenosine	$C_{11}H_{15}N_5O_3S$	13.86	296.09	26.63	0.25	26.11	0.08	25.59	0.27	26.12	0.09	24.72	0.06	ko01100; ko00270; ko00908
LPG 16:0	$C_{22}H_{45}O_9P$	14.27	483.27	27.30	0.34	29.77	0.04	27.93	0.39	28.32	0.14	28.28	0.17	
Corchorifatty acid F	$C_{18}H_{32}O_5$	10.94	327.22	23.70	0.18	24.07	0.15	24.17	0.13	24.17	0.03	24.47	0.08	
Prostaglandin H2	$C_{20}H_{32}O_5$	12.87	333.21	21.96	0.16	22.10	0.04	22.22	1.44	22.62	0.20	21.52	0.25	ko01100; ko00590
Myristoleic Acid	$C_{14}H_{26}O_2$	13.80	225.19	27.06	0.17	25.21	0.13	26.56	0.28	26.15	0.09	26.12	0.06	
(±)9(10)-EpOME	$C_{18}H_{32}O_3$	11.71	341.23	20.65	0.30	21.58	0.09	21.76	0.17	21.58	0.27	21.64	0.15	
LPI 16:1	$C_{25}H_{47}O_{12}P$	13.86	569.27	22.54	0.46	24.05	0.06	23.02	0.19	24.05	0.06	23.92	0.13	
S-Adenosyl-L-homocysteine	$C_{14}H_{20}N_6O_5S$	9.65	383.10	18.02	0.24	22.14	0.05	18.34	0.05	20.86	0.04	20.37	0.28	ko01100; ko01230; ko00270
13,14-Dihydro-15-keto-tetranor prostaglandin F1α	$C_{16}H_{28}O_5$	10.13	299.19	20.87	0.31	19.36	0.16	21.32	0.43	20.63	0.18	20.18	0.05	
8(S)-Hydroxy-(5Z,9E,11Z,14Z)-	$C_{20}H_{32}O_3$	13.55	319.23	23.92	0.25	22.67	0.13	21.49	0.27	23.61	0.14	21.89	0.33	ko01100;

eicosatetraenoic acid													ko00590
Phenobarbital-d5	$C_{12}H_7H_5N_2O_3$	12.82	236.11	19.22	0.23	20.37	0.05	20.13	0.26	20.66	0.11	23.07	0.07	
Neocnidilide	$C_{12}H_{18}O_2$	9.63	193.12	20.15	0.12	21.37	0.16	21.73	0.15	21.04	0.02	22.20	0.07	
Quercetin	$C_{15}H_{10}O_7$	8.57	301.04	22.16	0.47	23.00	0.12	21.95	0.20	23.28	0.09	23.85	0.14	ko01100; ko01110; ko00941; ko00944
PC (16:0e/2:0)	$C_{26}H_{54}NO_7P$	12.75	568.35	16.97	0.27	19.28	0.29	18.39	0.54	17.12	0.08	17.58	0.90	
DGDG (8:0/8:0)	$C_{31}H_{56}O_{15}$	14.25	713.36	19.15	0.25	18.84	0.03	19.06	0.10	20.00	0.90	19.44	0.07	
Trehalose-6-phosphate	$C_{12}H_{23}O_{14}P$	1.37	421.07	19.42	0.05	21.43	0.46	19.44	0.11	19.96	0.21	19.22	0.18	ko01100; ko00500
Corticosterone	$C_{21}H_{30}O_4$	12.49	345.21	20.42	0.46	18.68	0.10	18.68	0.40	20.59	0.09	20.01	0.31	ko01100
Poncirin	$C_{28}H_{34}O_{14}$	13.87	273.08	21.65	0.19	21.12	0.30	20.09	0.41	20.38	0.07	18.04	0.43	
Tretinoin	$C_{20}H_{28}O_2$	14.09	299.20	25.51	0.35	24.79	0.31	23.69	0.13	26.17	0.30	24.72	0.35	
2,3-dinor Prostaglandin E1	$C_{18}H_{30}O_5$	12.14	307.19	22.42	0.28	24.54	0.03	23.79	0.33	23.05	0.06	22.92	0.01	
Didymin	$C_{28}H_{34}O_{14}$	13.86	593.18	25.92	0.65	26.20	0.13	25.76	0.29	26.04	0.10	24.56	0.05	

Note: The values in the table are relative quantitative values without units.

Supplementary Table S2. Key differential metabolites Log_2 fold-change in POD activity

Name	Formula	RT [min]	m/z	Control group-0h		200 mM NaCl-48h		200 mM NaCl + 10 mM KCl-48h		200 mM NaCl-168h		200 mM NaCl + 10 mM KCl-168h		Pathway
				Mean	Standard deviation	Mean	Standard deviation	Mean	Standard deviation	Mean	Standard deviation	Mean	Standard deviation	
**Positive**														
N-Acetyltryptophan	$C_{13}H_{14}N_2O_3$	8.99	247.11	26.98	0.03	26.01	0.11	26.37	0.03	26.12	0.05	26.73	0.05	
ARH	$C_{15}H_{26}N_8O_4$	12.69	383.22	26.64	0.02	23.15	0.35	26.59	0.20	24.88	0.18	26.83	0.11	
N2-Acetyl-L-ornithine	$C_7H_{14}N_2O_3$	8.82	175.11	23.71	0.44	26.69	0.07	24.33	0.21	26.49	0.25	23.30	0.34	ko01100; ko01110; ko01130; ko01230; ko01210; ko00220
2-Methoxyresorcinol	$C_7H_8O_3$	6.20	141.05	22.96	0.33	26.54	0.15	23.63	0.09	23.03	0.13	24.02	0.07	
Oryzalexin E	$C_{20}H_{32}O_2$	15.06	305.25	22.33	0.16	23.28	0.32	21.90	0.22	23.63	0.28	22.08	0.12	ko01110
DL-Tryptophan	$C_{11}H_{12}N_2O_2$	6.78	205.10	30.17	0.08	28.31	0.45	29.82	0.06	28.48	0.01	30.04	0.02	
1,2,3,9-tetrahydro-4H-carbazol-4-one oxime	$C_{12}H_{12}N_2O$	8.99	201.10	26.84	0.02	25.82	0.32	26.33	0.05	26.00	0.02	26.65	0.03	
Tranilast	$C_{18}H_{17}NO_5$	8.41	328.12	24.69	0.12	26.45	0.03	24.87	0.14	24.57	0.10	23.28	0.26	
Eugenol	$C_{10}H_{12}O_2$	6.56	165.09	26.34	0.05	24.12	0.04	26.05	0.01	25.82	0.05	26.43	0.07	ko00940
15-Deoxy-Δ12,14-prostaglandin A1	$C_{20}H_{30}O_3$	14.38	301.22	20.67	0.32	19.67	0.12	19.80	0.29	21.75	0.20	20.50	0.38	
p-Coumaric acid	$C_9H_8O_3$	6.26	165.05	24.52	0.24	23.68	0.19	24.61	0.15	23.91	0.06	24.82	0.21	ko01100; ko01110; ko00940; ko00360; ko00950; ko01220; ko00350; ko00130
1,4-dihydroxyheptadec-16-en-2-yl acetate	$C_{19}H_{36}O_4$	14.60	351.25	25.39	0.51	23.30	0.05	24.30	0.65	25.56	0.27	25.11	0.04	
8-Hydroxyquinoline	C_9H_7NO	8.93	146.06	25.66	0.37	25.89	0.12	26.05	0.07	24.89	0.18	25.69	0.06	
4-(4-methylphenyl)-2-phenyl-1,3-	$C_{16}H_{13}NS$	8.19	252.09	25.47	0.10	24.88	0.07	25.74	0.10	25.45	0.07	25.37	0.13	

thiazole														
3-Methylcrotonylglycine	$C_7H_{11}NO_3$	7.00	158.08	26.11	0.04	25.54	0.04	26.02	0.08	25.41	0.08	25.57	0.11	
Kahweol	$C_{20}H_{26}O_3$	12.99	315.19	20.83	0.48	20.41	0.30	19.53	0.25	20.52	0.31	19.94	0.20	
3-(4-hydroxy-3-methoxyphenyl)propanoic acid	$C_{10}H_{12}O_4$	9.28	179.07	23.71	0.06	23.63	0.25	23.34	0.09	25.21	0.02	25.03	0.27	
Norverapamil	$C_{26}H_{36}N_2O_4$	12.34	441.28	24.77	0.07	21.13	0.30	25.05	0.10	24.19	0.05	25.75	0.09	
2-Hydroxyphenylalanine	$C_9H_{11}NO_3$	1.98	182.08	25.69	0.11	24.32	0.12	25.24	0.53	24.37	0.04	25.37	0.39	
7-Methoxy-4-methylcoumarin	$C_{11}H_{10}O_3$	9.58	191.07	23.54	0.12	24.55	0.06	23.56	0.30	24.57	0.10	23.10	0.39	
Bavachin	$C_{20}H_{20}O_4$	2.43	325.14	25.33	0.20	23.05	0.49	25.12	0.07	25.25	0.08	25.49	0.11	
4-oxo-4,5,6,7-tetrahydrobenzo[b]furan-3-carboxylic acid	$C_9H_8O_4$	7.70	181.05	23.85	0.31	23.82	0.22	23.80	0.30	25.46	0.09	23.72	0.07	
3-phenyl-5-(trifluoromethyl)-4,5-dihydro-1H-pyrazol-5-ol	$C_{10}H_9F_3N_2O$	8.99	231.08	25.54	0.26	24.69	0.31	25.18	0.06	24.92	0.02	25.49	0.06	
2-[(2,3,4,5,6-pentamethylbenzyl)thio]-4,5-dihydro-1,3-thiazole	$C_{15}H_{21}NS_2$	10.31	280.12	21.10	0.19	22.27	0.25	20.82	0.07	24.57	0.05	22.21	0.13	
methyl 2-(2-acetyl-4,5-dimethoxyphenyl)acetate	$C_{13}H_{16}O_5$	10.27	253.11	21.40	0.10	24.25	0.49	22.47	0.55	24.36	0.06	22.94	0.41	
Indole	C_8H_7N	8.99	118.07	25.47	0.11	24.64	0.14	25.10	0.08	24.60	0.07	25.19	0.11	ko01100; ko01110; ko00400; ko00380; ko00402
Isotretinoin	$C_{20}H_{28}O_s$	14.65	301.22	22.83	0.22	23.21	0.32	21.56	0.17	24.43	0.15	22.57	0.17	
D-(+)-Proline	$C_5H_9NO_2$	1.35	116.07	29.67	0.14	31.86	0.03	31.51	0.04	31.51	0.02	31.42	0.05	
PEtOH (16:2-18:2)	$C_{39}H_{69}O_8P$	14.65	714.51	21.90	0.68	24.84	0.36	20.64	0.22	22.13	0.02	21.14	0.77	
Diaminopimelic acid	$C_7H_{14}N_2O_4$	1.34	174.08	24.09	0.05	22.73	0.18	23.34	0.06	24.07	0.09	23.05	0.22	ko01100; ko01110; ko01120; ko01130; ko01230; ko00300
BMP (6:0/24:2)	$C_{36}H_{67}O_{10}P$	14.29	708.47	20.09	0.59	24.83	0.10	19.20	0.99	19.64	0.80	18.96	1.00	

2-{[(4,5-dimethoxy-2-nitrophenethyl)imino]methyl}phenol	$C_{17}H_{18}N_2O_5$	9.52	331.14	24.54	0.33	23.70	0.16	24.21	0.39	24.46	0.05	25.10	0.25	
Picropodophyllotoxin	$C_{22}H_{22}O_8$	1.34	432.17	24.61	0.15	23.50	0.09	25.05	0.20	23.00	0.21	23.37	0.24	
PE (6:0/21:1)	$C_{32}H_{62}NO_8P$	14.21	620.44	21.95	0.23	25.10	0.06	21.03	0.28	21.29	0.28	20.44	0.42	
2-chloro-N-[4-(4-methylpiperazino)phenyl]benzamide	$C_{18}H_{20}ClN_3O$	8.96	330.13	30.25	0.06	31.66	0.10	30.56	0.06	30.91	0.04	30.51	0.05	
GRH	$C_{14}H_{24}N_8O_4$	12.13	369.20	28.68	0.04	24.41	0.33	28.84	0.08	27.42	0.09	28.92	0.14	
Valepotriate	$C_{22}H_{30}O_8$	9.35	423.20	25.14	0.20	22.86	0.27	23.43	0.18	24.75	0.01	24.55	0.29	
2-(2-thienyl)-1,3-thiazole-4-carboxylic acid	$C_8H_5NO_2S_2$	1.10	249.94	24.66	0.09	23.80	0.14	24.42	0.25	23.33	0.33	24.93	0.19	
HPK	$C_{17}H_{28}N_6O_4$	12.79	381.22	24.95	0.26	20.43	1.12	24.67	0.18	22.54	0.71	24.76	0.28	
5-[(10Z)-14-(3,5-dihydroxyphenyl)tetradec-10-en-1-yl]benzene-1,3-diol	$C_{26}H_{36}O_4$	14.37	435.25	24.78	0.18	23.15	0.17	24.99	0.10	23.73	0.14	24.76	0.13	
BMP (6:0/26:2)	$C_{38}H_{71}O_{10}P$	14.58	736.52	21.41	0.37	24.83	0.15	21.13	0.29	22.16	0.35	20.24	0.35	
DGMG (18:2)	$C_{33}H_{58}O_{14}$	14.27	679.39	28.82	0.14	28.04	0.13	28.40	0.10	28.31	0.11	28.89	0.09	
15-Acetyldeoxynivalenol	$C_{17}H_{22}O_7$	10.38	321.13	21.08	0.17	22.35	0.16	21.10	0.10	22.86	0.04	21.17	0.15	
13-HPODE	$C_{18}H_{32}O_4$	11.84	313.24	23.88	0.16	20.53	0.08	24.44	0.08	23.05	0.03	24.88	0.07	ko01100; ko00591
Isoquinoline	C_9H_7N	8.99	130.07	28.95	0.02	28.02	0.08	28.42	0.07	28.13	0.03	28.70	0.08	
17α-Ethynylestradiol	$C_{20}H_{24}O_2$	12.24	297.18	20.68	0.75	19.65	0.08	20.19	0.08	20.74	0.32	20.21	0.87	
Miquelianin	$C_{21}H_{18}O_{13}$	9.20	479.08	26.00	0.15	28.53	0.03	26.47	0.29	27.72	0.11	27.74	0.06	
7-(2-hydroxypropan-2-yl)-1,4a-dimethyl-decahydronaphthalen-1-ol	$C_{15}H_{28}O_2$	13.47	223.21	22.54	0.08	20.77	0.50	20.88	0.18	23.64	0.30	19.94	0.16	
3-Methoxytyramine	$C_9H_{13}NO_2$	3.83	168.10	27.16	0.48	24.58	0.11	27.75	0.16	25.76	0.01	28.68	0.04	ko01100; ko00350; ko00965
N-morpholino-N'-[2-(trifluoromethyl)benzoyl]urea	$C_{13}H_{14}F_3N_3O_3$	11.78	318.10	25.24	0.29	22.51	0.14	23.16	0.32	28.48	0.13	24.37	0.14	
Heptadecanoic Acid	$C_{17}H_{34}O_2$	13.31	293.25	22.75	0.11	22.31	0.10	22.08	0.04	24.24	0.16	22.50	0.03	
Calceolarioside B	$C_{23}H_{26}O_{11}$	8.81	479.15	23.55	0.13	28.10	0.22	25.20	0.54	25.56	0.03	25.25	0.18	
Asp-Glu	$C_9H_{14}N_2O_7$	1.36	263.09	23.68	0.05	23.89	0.27	23.78	0.58	22.88	0.12	24.40	0.09	

Eicosapentaenoic acid	$C_{20}H_{30}O_2$	13.94	303.23	23.84	0.05	24.05	0.20	23.94	0.31	25.00	0.32	23.38	0.43	ko01040
Indole-3-acrylic acid	$C_{11}H_9NO_2$	8.99	188.07	28.41	0.05	27.51	0.09	27.94	0.12	27.63	0.00	28.21	0.10	
6,15-diketo-13,14-dihydro Prostaglandin F1α	$C_{20}H_{34}O_6$	12.13	353.23	30.43	0.02	27.48	0.30	30.78	0.10	30.77	0.04	31.39	0.10	
Tyramine	$C_8H_{11}NO$	2.40	138.09	28.14	0.22	25.51	0.07	27.75	0.19	27.79	0.14	28.16	0.10	ko01110; ko00950; ko00350
Phloretin	$C_{15}H_{14}O_5$	7.68	275.09	24.74	0.08	27.03	0.16	25.67	0.22	25.95	0.21	24.86	0.07	ko01110; ko00941
Adenosine	$C_{10}H_{13}N_5O_4$	2.86	268.10	27.23	0.17	24.31	0.99	27.73	0.26	22.74	2.89	27.93	0.16	ko01100; ko00230
Mevalonic acid	$C_6H_{12}O_4$	6.61	149.08	21.76	0.10	23.94	0.05	24.01	0.16	21.48	0.13	21.50	0.14	ko01100; ko01110; ko01130; ko00900
Caffeic acid	$C_9H_8O_4$	7.45	181.05	25.57	0.12	27.78	0.10	26.92	0.41	24.95	0.05	25.87	0.08	
4-chloro-N-(2-morpholinophenyl)benzamide	$C_{17}H_{17}ClN_2O_2$	12.03	317.10	25.78	0.30	22.68	0.36	22.92	0.57	27.66	0.06	23.10	0.20	
9-Oxo-ODE	$C_{18}H_{30}O_3$	12.13	295.23	30.36	0.09	28.06	0.40	30.49	0.19	30.47	0.02	30.77	0.05	ko00591
1-(3-acetyl-2,4,6-trihydroxyphenyl)ethan-1-one	$C_{10}H_{10}O_5$	7.88	211.06	27.41	0.04	25.75	0.05	27.26	0.06	26.19	0.05	27.36	0.10	
Syringic acid	$C_9H_{10}O_5$	7.73	199.06	26.00	0.10	25.45	0.11	25.37	0.12	27.28	0.04	26.09	0.07	ko01120
PC (14:1e/2:0)	$C_{24}H_{48}NO_7P$	14.26	494.32	26.71	0.24	25.74	0.16	26.52	0.19	26.55	0.14	25.77	0.13	
3-Methoxybenzaldehyde	$C_8H_8O_2$	6.81	137.06	26.05	0.10	25.33	0.16	26.05	0.26	26.39	0.07	25.67	0.02	
Sedanolide	$C_{12}H_{18}O_2$	12.13	177.13	26.67	0.02	23.87	0.30	26.73	0.15	26.55	0.11	26.88	0.13	
N-[(4-hydroxy-3-methoxyphenyl)methyl]-8-methylnonanamide	$C_{18}H_{29}NO_3$	13.47	308.22	24.57	0.11	25.48	0.41	24.29	1.00	25.99	0.27	23.35	0.16	
**Negative**														
Glycerophospho-N-palmitoyl ethanolamine	$C_{21}H_{44}NO_7P$	14.70	452.28	29.39	0.18	27.09	0.09	29.54	0.09	27.96	0.03	29.65	0.18	
L-Lysine	$C_6H_{14}N_2O_2$	1.84	145.10	25.70	0.29	22.31	0.05	25.91	0.11	24.33	0.08	25.63	0.10	ko01100; ko01110 ;

														ko01120; ko01130; ko01230; ko02010; ko01210; ko00960; ko00970; ko00300; ko00310; ko00780
PA (2:0/20:1)	$C_{25}H_{47}O_8P$	14.23	505.30	23.87	0.51	26.76	0.09	24.06	0.30	24.38	0.08	24.54	0.06	
LPS 16:0	$C_{22}H_{44}NO_9P$	14.36	496.27	24.81	0.21	22.49	0.25	24.72	0.18	24.34	0.10	25.99	0.21	
LPA 9:0	$C_{12}H_{25}O_7P$	1.32	311.12	23.11	0.52	21.74	1.80	23.89	0.06	25.19	0.31	26.00	0.12	
Nepodin	$C_{13}H_{12}O_3$	1.35	215.07	24.71	0.14	23.03	0.28	25.55	0.32	21.01	0.26	22.96	0.67	
PI (2:0/22:2)	$C_{33}H_{59}O_{13}P$	13.05	693.37	24.29	0.20	23.25	0.07	24.95	0.23	24.16	0.02	25.83	0.22	
Timosaponin A-III	$C_{39}H_{64}O_{13}$	14.56	739.41	25.42	0.28	22.57	0.10	24.81	0.26	24.77	0.07	25.19	0.13	
2'-Deoxyinosine 5'-monophosphate	$C_{10}H_{13}N_4O_7P$	8.93	331.05	25.77	0.15	27.93	0.05	26.06	0.13	26.53	0.10	26.16	0.03	ko00230
PA (2:0/19:1)	$C_{24}H_{45}O_8P$	10.86	491.29	27.28	0.15	22.61	0.27	27.96	0.21	26.50	0.14	28.91	0.07	
LPI 16:0	$C_{25}H_{49}O_{12}P$	14.33	571.29	29.23	0.26	26.77	0.08	28.90	0.13	28.03	0.08	29.19	0.20	
N-(4-bromo-1-methyl-1H-pyrazol-5-yl)-2,2-dimethylpropanamide	$C_9H_{14}BrN_3O$	7.77	258.02	22.16	0.34	25.02	0.07	23.55	0.55	20.97	0.15	22.51	0.23	
LPG 18:1	$C_{24}H_{47}O_9P$	14.39	509.29	26.91	0.58	26.81	0.22	26.98	0.15	27.81	0.31	26.22	0.15	
Pizotifen	$C_{19}H_{21}NS$	13.49	294.14	18.59	0.66	16.93	0.22	18.71	0.39	27.20	0.25	25.82	0.08	
(+/-)11(12)-EET	$C_{20}H_{32}O_3$	13.35	319.23	23.25	0.20	22.87	0.23	22.82	0.61	23.69	0.09	22.38	0.16	
Norcimifugin	$C_{15}H_{16}O_6$	7.85	291.09	23.86	0.01	26.06	0.11	24.62	0.02	26.48	0.06	23.67	0.21	
Ganoderic acid C6	$C_{30}H_{42}O_8$	13.76	529.29	19.25	1.65	20.72	0.14	23.96	0.30	26.34	0.01	24.03	0.02	
4-Acetamidobutanoic acid	$C_6H_{11}NO_3$	1.34	144.07	28.74	0.05	28.60	0.48	29.11	0.44	29.73	0.37	31.16	0.21	ko01100; ko00330
D-(-)-Quinic acid	$C_7H_{12}O_6$	1.23	191.06	27.03	0.14	26.71	0.15	26.93	0.27	26.81	0.08	27.39	0.16	
Nonanoic acid	$C_9H_{18}O_2$	12.01	157.12	26.50	0.30	25.86	0.06	26.24	0.03	26.19	0.10	26.59	0.21	
Berberine	$C_{20}H_{18}NO_4$	8.73	335.12	23.78	0.42	18.72	0.06	17.15	0.09	25.69	0.08	19.73	0.51	ko01110; ko00950
5,6-dimethyl-3-[5-(trifluoromethyl)pyridin-2-yl]-1,2,4-	$C_{11}H_9F_3N_4$	6.60	253.08	23.53	0.22	19.65	0.07	19.91	0.04	25.63	0.03	21.52	0.18	

triazine														
α,α-Trehalose	$C_{12}H_{22}O_{11}$	1.36	341.11	31.36	0.20	32.47	0.15	32.08	0.37	27.92	0.09	29.64	0.71	
(±)9-HpODE	$C_{18}H_{32}O_4$	12.05	293.21	25.78	0.12	23.09	0.38	26.20	0.45	26.52	0.06	27.38	0.08	
(+/-)18-HEPE	$C_{20}H_{30}O_3$	13.02	317.21	21.37	0.30	24.30	0.22	21.52	0.15	24.22	0.14	22.80	0.09	
PA (2:0/16:4)	$C_{21}H_{33}O_8P$	12.32	443.18	23.04	0.29	20.25	0.16	19.77	0.21	22.61	0.10	24.01	0.05	
Arachidonic acid	$C_{20}H_{32}O_2$	14.47	303.23	27.56	0.29	27.73	0.41	26.20	0.18	28.26	0.08	27.04	0.19	ko01100; ko01040; ko00590; ko00591
16-Hydroxyhexadecanoic acid	$C_{16}H_{32}O_3$	14.33	253.22	31.48	0.19	29.87	0.32	30.23	0.20	30.70	0.10	30.14	0.18	
cis-5,8,11,14,17-Eicosapentaenoic acid	$C_{20}H_{30}O_2$	14.20	301.22	25.74	0.38	28.70	0.43	25.81	0.20	28.40	0.13	27.46	0.15	ko01040
2,3-dihydro-2-spiro[1-(benzyl)piperidin-4-yl]-1,3-benzothiazole	$C_{18}H_{20}N_2S$	9.82	295.12	27.61	0.17	17.38	0.53	18.35	0.32	29.53	0.04	25.32	0.13	
2-[6-(1H-benzo[d]imidazol-2-yl)-2-pyridyl]-1H-benzo[d]imidazole	$C_{19}H_{13}N_5$	8.33	311.12	24.81	0.16	20.10	0.19	20.02	0.10	27.10	0.05	22.08	0.18	
Mycophenolic acid	$C_{17}H_{20}O_6$	10.13	319.12	24.45	0.20	21.04	0.12	20.10	0.04	26.22	0.01	22.08	0.13	
LPG 18:3	$C_{24}H_{43}O_9P$	13.82	505.26	24.17	0.41	25.77	0.35	25.22	0.04	24.95	0.15	24.59	0.21	
LysoPE 18:2 (2n isomer)	$C_{23}H_{44}NO_7P$	14.09	476.28	28.04	0.25	25.63	0.18	27.82	0.22	26.70	0.06	28.41	0.11	
Prostaglandin H2	$C_{20}H_{32}O_5$	12.87	333.21	21.96	0.16	22.10	0.04	22.22	1.44	22.62	0.20	21.52	0.25	ko01100; ko00590
Myristoleic Acid	$C_{14}H_{26}O_2$	13.80	225.19	27.06	0.17	25.21	0.13	26.56	0.28	26.15	0.09	26.12	0.06	
LPI 18:3	$C_{27}H_{47}O_{12}P$	13.77	593.27	26.68	0.28	26.78	0.22	26.84	0.04	27.43	0.15	26.43	0.19	
LPI 16:1	$C_{25}H_{47}O_{12}P$	13.86	569.27	22.54	0.46	24.05	0.06	23.02	0.19	24.05	0.06	23.92	0.13	
Pristimerin	$C_{30}H_{40}O_4$	11.73	463.29	26.16	0.16	22.74	0.08	27.02	0.24	26.18	0.11	27.42	0.04	
Flavin mononucleotide (FMN)	$C_{17}H_{21}N_4O_9P$	7.75	455.10	23.63	0.18	25.98	0.14	24.48	0.50	22.72	0.02	23.31	0.19	
L-Tryptophan	$C_{11}H_{12}N_2O_2$	6.01	203.08	27.23	0.32	25.05	0.06	26.80	0.18	25.45	0.14	27.55	0.16	ko01100; ko01110; ko01130; ko01230; ko01210; ko00400; ko00260;

																ko00380; ko00970; ko00901; ko00966
Dodecanedioic acid	$C_{12}H_{22}O_4$	11.18	229.15	24.91	0.07	21.40	0.22	25.18	0.16	24.76	0.09	25.66	0.06			
di-C,C-pentosyl-apigenin	$C_{25}H_{26}O_{13}$	13.58	533.13	18.14	0.16	27.02	0.07	18.04	0.18	18.09	0.04	18.30	0.10			
8(S)-Hydroxy-(5Z,9E,11Z,14Z)-eicosatetraenoic acid	$C_{20}H_{32}O_3$	13.55	319.23	23.92	0.25	22.67	0.13	21.49	0.27	23.61	0.14	21.89	0.33			ko01100; ko00590
LPC(1-acyl 16:0)	$C_{24}H_{50}NO_7P$	14.76	494.33	25.50	0.32	22.63	0.16	25.20	0.83	24.10	0.17	26.27	0.20			
Phenobarbital-d5	$C_{12}H_7H_5N_2O_3$	12.82	236.11	19.22	0.23	20.37	0.05	20.13	0.26	20.66	0.11	23.07	0.07			
Cichoric acid	$C_{22}H_{18}O_{12}$	1.36	473.07	25.10	0.20	25.28	0.23	26.18	0.32	22.44	0.44	23.83	0.90			
2,4,6-Trihydroxyacetophenone	$C_8H_8O_4$	7.46	167.04	24.98	0.11	26.28	0.11	24.39	1.00	25.91	0.03	24.91	0.21			
GlcADG (16:0-16:2)	$C_{41}H_{72}O_{11}$	14.00	739.50	25.75	0.18	23.69	0.26	24.50	0.08	25.36	0.04	25.34	0.07			
Raffinose	$C_{18}H_{32}O_{16}$	1.36	563.18	25.28	0.12	22.91	0.22	24.86	0.22	21.42	0.19	24.62	0.35			ko02010; ko00052
Eupatilin	$C_{18}H_{16}O_7$	10.99	343.08	25.91	0.42	26.19	0.03	23.75	0.79	26.49	0.04	24.36	0.34			
LPC 18:2	$C_{26}H_{50}NO_7P$	14.42	518.32	27.32	0.46	25.87	0.20	27.40	0.20	26.60	0.13	27.45	0.29			
LPC 16:0	$C_{24}H_{50}NO_7P$	14.76	554.35	28.19	0.27	25.92	0.08	27.72	0.83	26.93	0.15	28.37	0.28			
Methyl gallate	$C_8H_8O_5$	7.25	183.03	29.01	0.10	28.23	0.41	28.88	0.04	27.38	0.04	28.23	0.20			
Pyrogallol	$C_6H_6O_3$	1.38	125.02	25.56	0.07	24.84	0.26	25.92	0.03	24.97	0.04	25.95	0.20			ko01120
Dehydroascorbic acid	$C_6H_6O_6$	1.40	173.01	25.87	0.11	25.61	0.05	25.80	0.03	25.86	0.04	26.68	0.07			ko01100; ko00053; ko00480
5-Methoxypsoralen	$C_{12}H_8O_4$	1.40	215.03	28.85	0.22	28.72	0.35	30.40	0.33	29.74	0.53	30.88	0.15			ko01110
Sinapinic acid	$C_{11}H_{12}O_5$	6.05	223.06	26.18	0.42	19.24	0.28	23.84	0.31	23.46	0.31	25.71	0.10			ko01100; ko01110; ko00940
D-(-)-Lyxose	$C_5H_{10}O_5$	1.29	149.05	25.56	0.14	24.85	0.10	25.87	0.07	25.77	0.27	26.42	0.07			
Baicalin methyl ester	$C_{22}H_{20}O_{11}$	1.35	459.09	21.28	0.38	24.02	0.34	25.15	0.32	21.14	0.46	23.22	1.03			
N4-(5-chloro-4-methoxy-3-thienyl)-2,6-dimethylmorpholine-4-carboxamide	$C_{12}H_{17}ClN_2O_3S$	7.46	303.06	24.67	0.16	21.53	0.02	22.79	0.12	25.88	0.07	25.47	0.07			
D-myo-Inositol 1,4-bisphosphate	$C_6H_{14}O_{12}P_2$	1.11	338.99	24.52	0.14	23.89	0.20	26.15	0.06	24.23	0.15	26.70	0.11			ko01100; ko00562 ;

														ko04070
Corticosterone	$C_{21}H_{30}O_4$	12.49	345.21	20.42	0.46	18.68	0.10	18.68	0.40	20.59	0.09	20.01	0.31	ko01100
PA (18:3/22:6)	$C_{43}H_{67}O_8P$	14.27	741.44	25.02	0.08	22.09	0.33	24.58	0.11	25.53	0.08	25.45	0.07	
Coniferyl alcohol	$C_{10}H_{12}O_3$	8.55	179.07	23.80	0.28	22.65	0.27	23.17	0.17	22.13	0.13	24.51	0.08	ko01100; ko01110; ko00940
DGDG (7:0/9:0)	$C_{31}H_{56}O_{15}$	11.15	727.38	23.99	0.11	21.50	0.17	24.61	0.33	23.25	0.07	24.99	0.06	
D-Raffinose	$C_{18}H_{32}O_{16}$	1.36	503.16	27.06	0.10	25.23	0.21	27.14	0.14	24.26	0.11	27.20	0.23	ko02010; ko00052
Polyphyllin VI	$C_{39}H_{62}O_{13}$	14.26	737.40	27.89	0.21	27.64	0.13	27.44	0.10	27.34	0.19	28.00	0.12	
Tretinoin	$C_{20}H_{28}O_2$	14.09	299.20	25.51	0.35	24.79	0.31	23.69	0.13	26.17	0.30	24.72	0.35	
PA (18:3/22:5)	$C_{43}H_{69}O_8P$	14.24	743.45	25.67	0.35	22.76	0.16	25.12	0.20	26.57	0.06	26.14	0.09	

Note: The values in the table are relative quantitative values without units.

Supplementary Table S3. Key differential metabolites Log_2 fold-change in CAT activity

Name	Formula	RT [min]	m/z	CK-0h		NaCl-48h		NaCl+KCl-48h		NaCl-168h		NaCl+KCl-168h		Pathway
				Average quantitative	Standard deviation	Average quantitative	Standard deviation	Average quantitative	Standard deviation	Average quantitative	Standard deviation	Average quantitative	Standard deviation	
**Positive**														
Oryzalexin E	$C_{20}H_{32}O_2$	15.06	305.25	22.33	0.16	23.28	0.32	21.90	0.22	23.63	0.28	22.08	0.12	ko01110
PC (18:5e/2:0)	$C_{28}H_{48}NO_7P$	14.11	542.32	23.40	0.30	23.86	0.31	22.81	0.16	22.96	0.07	23.09	0.95	
4-(4-methylphenyl)-2-phenyl-1,3-thiazole	$C_{16}H_{13}NS$	8.19	252.09	25.47	0.10	24.88	0.07	25.74	0.10	25.45	0.07	25.37	0.13	
PC (18:3e/2:0)	$C_{28}H_{52}NO_7P$	14.62	546.36	23.17	0.12	22.56	0.14	20.38	0.44	21.34	0.24	21.91	0.86	
LysoPC 18:0	$C_{26}H_{54}NO_7P$	14.59	546.35	23.12	0.12	22.58	0.07	20.31	0.46	21.23	0.38	21.87	0.85	
Isorhamnetin	$C_{16}H_{12}O_7$	9.53	317.07	22.21	0.47	26.88	0.15	25.94	0.27	26.34	0.19	26.87	0.70	
Eicosapentaenoic acid	$C_{20}H_{30}O_2$	13.94	303.23	23.84	0.05	24.05	0.20	23.94	0.31	25.00	0.32	23.38	0.43	ko01040
3,4-Methylenedioxy-α-pyrrolidinopropiophenone	$C_{14}H_{17}NO_3$	7.15	248.13	25.62	0.05	23.52	0.04	25.20	0.06	23.36	0.07	23.79	0.07	
**Negative**														
(+/-)11(12)-EET	$C_{20}H_{32}O_3$	13.35	319.23	23.25	0.20	22.87	0.23	22.816	0.607	23.69	0.09	22.38	0.16	
Arachidonic acid	$C_{20}H_{32}O_2$	14.47	303.23	27.56	0.29	27.73	0.41	26.201	0.176	28.26	0.08	27.04	0.19	ko01100; ko01040; ko00590; ko00591
16-Hydroxyhexadecanoic acid	$C_{16}H_{32}O_3$	14.33	253.22	31.48	0.19	29.87	0.32	30.232	0.197	30.70	0.10	30.14	0.18	
cis-5,8,11,14,17-Eicosapentaenoic acid	$C_{20}H_{30}O_2$	14.20	301.22	25.74	0.38	28.70	0.43	25.811	0.197	28.40	0.13	27.46	0.15	ko01040
Myristoleic Acid	$C_{14}H_{26}O_2$	13.80	225.19	27.06	0.17	25.21	0.13	26.563	0.279	26.15	0.09	26.12	0.06	
LPI 16:1	$C_{25}H_{47}O_{12}P$	13.86	569.27	22.54	0.46	24.05	0.06	23.019	0.195	24.05	0.06	23.92	0.13	
8(S)-Hydroxy-(5Z,9E,11Z,14Z)-eicosatetraenoic acid	$C_{20}H_{32}O_3$	13.55	319.23	23.92	0.25	22.67	0.13	21.487	0.266	23.61	0.14	21.89	0.33	ko01100; ko00590
Phenobarbital-d5	$C_{12}H_7H_5N_2O_3$	12.82	236.11	19.22	0.23	20.37	0.05	20.132	0.257	20.66	0.11	23.07	0.07	
Corticosterone	$C_{21}H_{30}O_4$	12.49	345.21	20.42	0.46	18.68	0.10	18.680	0.403	20.59	0.09	20.01	0.31	ko01100

Note: The values in the table are relative quantitative values without units.

Supplementary Table S4. Metabolite data analysis

Name	Formula	PPM	RT [min]	m/z	Control group (mean)	Standard deviation	200 mM NaCl 48h (mean)	Standard deviation	200 mM NaCl + 10 mM KCl 48h (mean)	Standard deviation	200 mM NaCl 168h (mean)	Standard deviation	200 mM NaCl + 10 mM KCl 168h (mean)	Standard deviation
Coniferyl alcohol	$C_{10}H_{12}O_3$	3.030653648	8.546	179.07199	14775495.44	3010746.476	6650075.125	1183618.112	9450893.614	1096536.468	4589796.763	424865.2427	23847671.96	1274536.728

Note: the metabolites data in the non-targeted metabolome detection are relative quantitative values without units.

Supplementary Table S5. Sequences of specific primers

ID	Primer name	Primer sequence (5'-3')
1	*Unigene0009260*	F: CCACTAGGTAGAAGAGACTCACTCAAGG
		R: TAAGGCAATCAAGTCGGCAACATCAA
2	*Unigene0024962*	F: GCTCCAACCAAAATGCGTCC
		R: CGGTTGGGCGTCTGGTCTTA
3	*Unigene0030384*	F: CGTCCCCTTGTTTCTGTGAG
		R: CATCTTTCACGACACTTTGG
4	*Unigene0033993*	F: GATCGGATCAACGGAGGCTCAATT
		R: GCTATAGACGGCGCATTCGGTAC
5	*Unigene0013825*	F: CTTGAGGACGAGGGGATTTG
		R: GTCGCACTTCCCCATCTTCT
6	*Unigene0084406*	F: GATGGAAACAAGACAACAGC
		R: GTTTTTCCTGATTTCTCCAC
7	*Unigene0019527*	F: TCGTGAATGACAAGGAAATC
		R: CAACTATCTGTCCATCATCG
8	*Unigene0053312*	F: GGAGTCACACCATTGGCGATTCA
		R: GGTTGCGTAATTCGGAAGCGTAGA
9	*Tubulin*	F: GCTGAGATTACAACCGCTG
		R: CTGTTCGTTTGGTCTTGATT

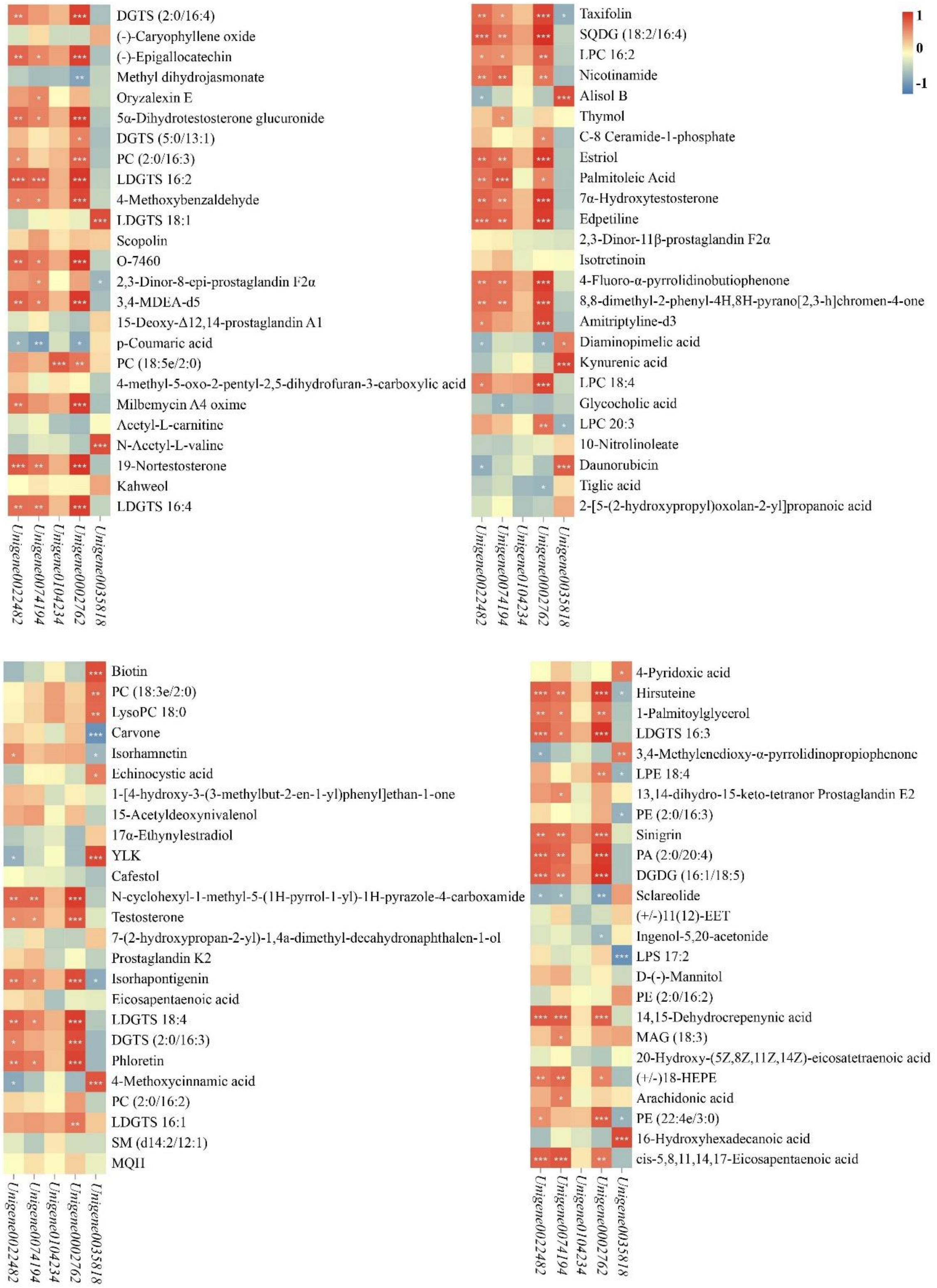

DGTS (2:0/16:4)
(-)-Caryophyllene oxide
(-)-Epigallocatechin
Methyl dihydrojasmonate
Oryzalexin E
5α-Dihydrotestosterone glucuronide
DGTS (5:0/13:1)
PC (2:0/16:3)
LDGTS 16:2
4-Methoxybenzaldehyde
LDGTS 18:1
Scopolin
O-7460
2,3-Dinor-8-epi-prostaglandin F2α
3,4-MDEA-d5
15-Deoxy-Δ12,14-prostaglandin A1
p-Coumaric acid
PC (18:5e/2:0)
4-methyl-5-oxo-2-pentyl-2,5-dihydrofuran-3-carboxylic acid
Milbemycin A4 oxime
Acetyl-L-carnitine
N-Acetyl-L-valine
19-Nortestosterone
Kahweol
LDGTS 16:4
Unigene0022482
Unigene0074194
Unigene0104234
Unigene0002762
Unigene0035818
Taxifolin
SQDG (18:2/16:4)
LPC 16:2
Nicotinamide
Alisol B
Thymol
C-8 Ceramide-1-phosphate
Estriol
Palmitoleic Acid
7α-Hydroxytestosterone
Edpetiline
2,3-Dinor-11β-prostaglandin F2α
Isotretinoin
4-Fluoro-α-pyrrolidinobutiophenone
8,8-dimethyl-2-phenyl-4H,8H-pyrano[2,3-h]chromen-4-one
Amitriptyline-d3
Diaminopimelic acid
Kynurenic acid
LPC 18:4
Glycocholic acid
LPC 20:3
10-Nitrolinoleate
Daunorubicin
Tiglic acid
2-[5-(2-hydroxypropyl)oxolan-2-yl]propanoic acid
1
0
-1
Biotin
PC (18:3e/2:0)
LysoPC 18:0
Carvone
Isorhamnetin
Echinocystic acid
1-[4-hydroxy-3-(3-methylbut-2-en-1-yl)phenyl]ethan-1-one
15-Acetyldeoxynivalenol
17α-Ethynylestradiol
YLK
Cafestol
N-cyclohexyl-1-methyl-5-(1H-pyrrol-1-yl)-1H-pyrazole-4-carboxamide
Testosterone
7-(2-hydroxypropan-2-yl)-1,4a-dimethyl-decahydronaphthalen-1-ol
Prostaglandin K2
Isorhapontigenin
Eicosapentaenoic acid
LDGTS 18:4
DGTS (2:0/16:3)
Phloretin
4-Methoxycinnamic acid
PC (2:0/16:2)
LDGTS 16:1
SM (d14:2/12:1)
MQII
4-Pyridoxic acid
Hirsuteine
1-Palmitoylglycerol
LDGTS 16:3
3,4-Methylenedioxy-α-pyrrolidinopropiophenone
LPE 18:4
13,14-dihydro-15-keto-tetranor Prostaglandin E2
PE (2:0/16:3)
Sinigrin
PA (2:0/20:4)
DGDG (16:1/18:5)
Sclareolide
(+/-)11(12)-EET
Ingenol-5,20-acetonide
LPS 17:2
D-(-)-Mannitol
PE (2:0/16:2)
14,15-Dehydrocrepenynic acid
MAG (18:3)
20-Hydroxy-(5Z,8Z,11Z,14Z)-eicosatetraenoic acid
(+/-)18-HEPE
Arachidonic acid
PE (22:4e/3:0)
16-Hydroxyhexadecanoic acid
cis-5,8,11,14,17-Eicosapentaenoic acid

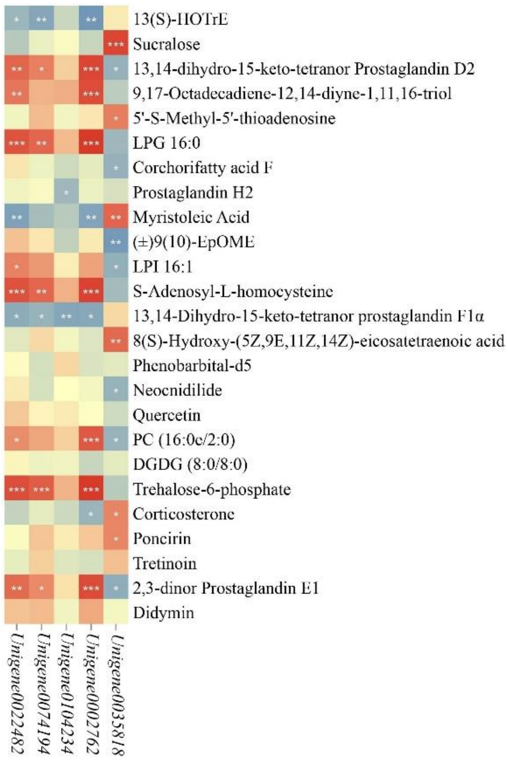

Supplementary Figure S1. Correlation heat map of DEGs and metabolites related to SOD activity in *T. ramosissima* (Heatmap of correlations between DEGs and metabolites in SOD activity. $p \geq 0.05$ is not marked; $0.01 < p < 0.05$ is marked as *; $0.001 < p < 0.01$ is marked as **; $p \leq 0.001$ is marked as ***).

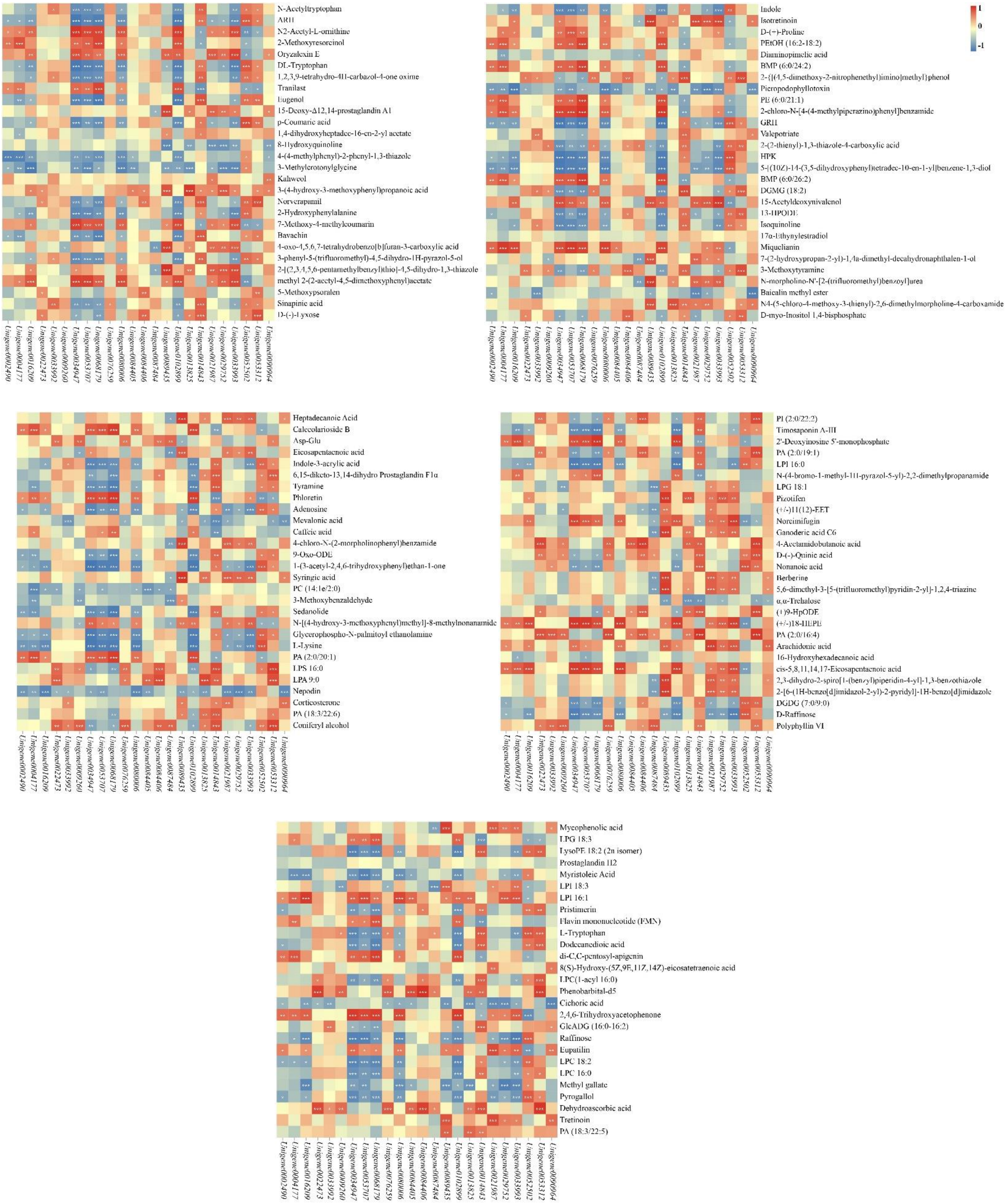

Supplementary Figure S2. Correlation heat map of DEGs and metabolites related to POD activity in *T. ramosissima* (Heatmap of correlations between DEGs and metabolites in POD activity. $p \geq 0.05$ is not marked; $0.01 < p < 0.05$ is marked as *; $0.001 < p < 0.01$ is marked as **; $p \leq 0.001$ is marked as ***).

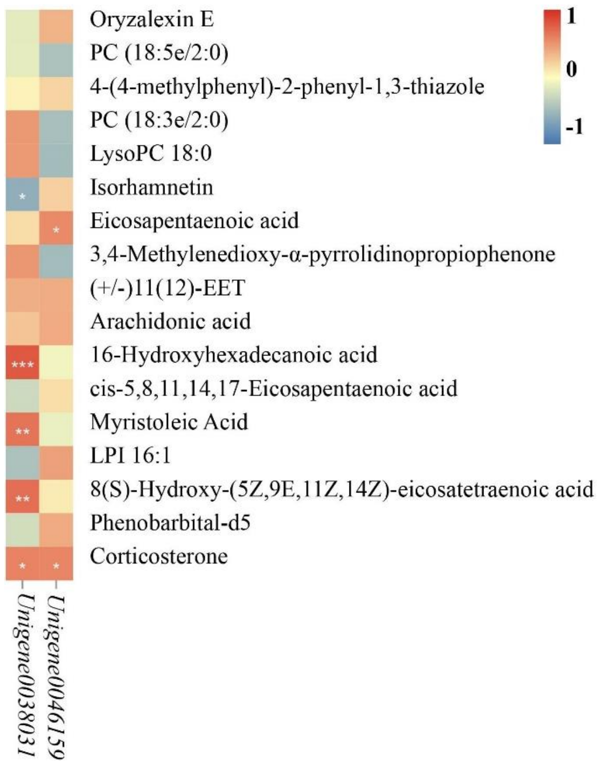

Supplementary Figure S3. Correlation heat map of DEGs and metabolites related to CAT activity in *T. ramosissima* (Heatmap of correlations between DEGs and metabolites in CAT activity. $p \geq 0.05$ is not marked; $0.01 < p < 0.05$ is marked as *; $0.001 < p < 0.01$ is marked as **; $p \leq 0.001$ is marked as ***).

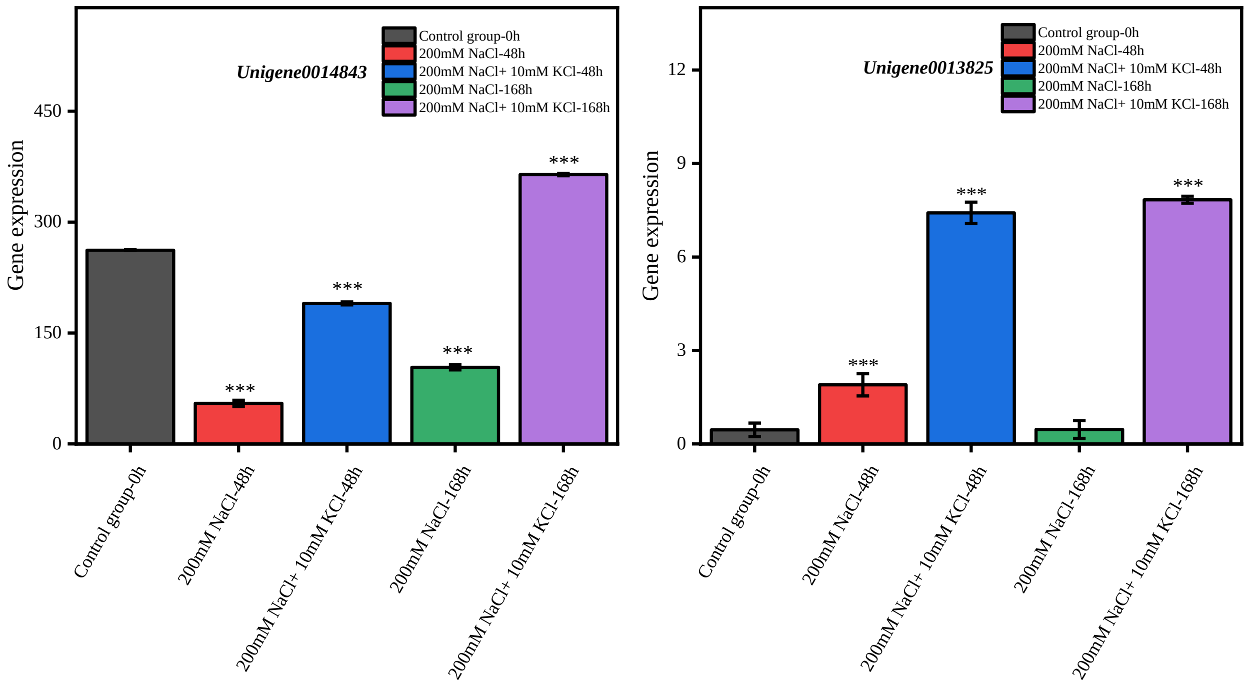

Supplementary Figure S4. Expression levels of *Unigene0014843* and *Unigene0013825* (*Unigene0014843* and *Unigene0013825* expression levels at 48h and 168h of 200 mM NaCl and 200 mM NaCl + 10 mM KCl treatment. Note: $p \geq 0.05$ is not marked; $0.01 < p < 0.05$ is marked as *; $0.001 < p < 0.01$ is marked as **; $p \leq 0.001$ is marked as ***; all of the data inside the figure are relative quantitative values, no unit required).

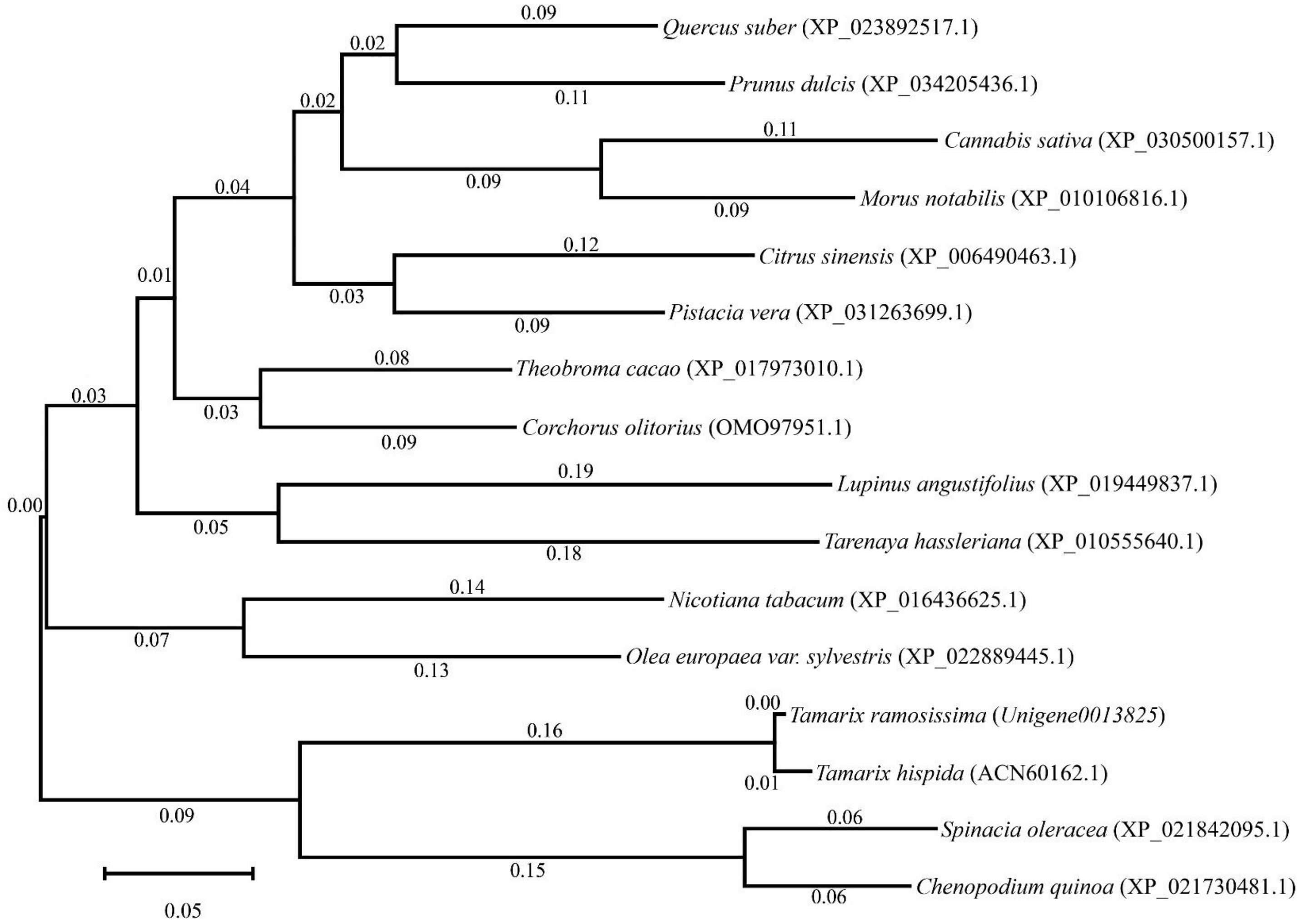

Supplementary Figure S5. Phylogenetic tree analysis of *T. ramosissima* POD and other species POD (A phylogenetic tree was constructed for analysis of the protein amino acid sequences of Unigene0013825 from *T. ramosissima* root and the protein amino acid sequences of other 15 species).
